# Enhancing cybersecurity in virtual power plants by detecting network based cyber attacks using an unsupervised autoencoder approach

**DOI:** 10.1038/s41598-025-01863-w

**Published:** 2025-09-05

**Authors:** Kumari Nutan Singh, Arup Kumar Goswami, Nalin Behari Dev Chudhury, Hassan Abdurrahman Shuaibu, Taha Selim Ustun

**Affiliations:** 1 Electrical Engineering Department, National Institute of Technology Silchar, Assam, 78801 India; 2 Department of Electrical, Telecommunications and Computer Engineering, Kampala International University, Kampala, Uganda; 3 Fukushima Renewable Energy Institute, National Institute of Advanced Industrial Science and Technology (AIST), Fukushima, 9630298 Koriyama Japan

**Keywords:** Virtual power plant (VPP), Renewable energy sources (RES), Energy market (EM), Cyber security, False data injection attack (FDIA), Auto-encoder (AE), Energy infrastructure, Renewable energy, Electrical and electronic engineering, Energy infrastructure

## Abstract

The increasing adoption of the Internet of Things (IoT) in energy systems has brought significant advancements but also heightened cyber security risks. Virtual Power Plants (VPPs), which aggregate distributed renewable energy resources into a single entity for participation in energy markets, are particularly vulnerable to cyber-attacks due to their reliance on modern information and communication technologies. Cyber-attacks targeting devices, networks, or specific goals can compromise system integrity. Common attack types include Denial of Service (DoS), Man-in-the-Middle (MITM), and False Data Injection Attacks (FDIA).Among these threats, FDIA are especially concerning as they manipulate critical operational data, such as bid prices and energy quantities, to disrupt system reliability, market stability, and financial performance. This study proposes an unsupervised Autoencoder (AE) deep learning approach to detect FDIA in VPP systems. The methodology is validated on a 9-bus and IEEE-39 bus test system modeled in MATLAB Simulink, encompassing renewable energy sources, energy storage systems, and variable loads. Time-series data generated over 1,000 days is used for training, validation, and testing the AE model. The results demonstrate the model’s ability to detect anomalies with high accuracy by analyzing reconstruction errors. By identifying false data, the approach ensures system reliability, protects against financial losses, and maintains energy market stability. This work highlights the importance of advanced machine learning techniques in enhancing cyber security for IoT-based energy systems and ensuring secure VPP operations.

## Introduction

**Fig. 1:**
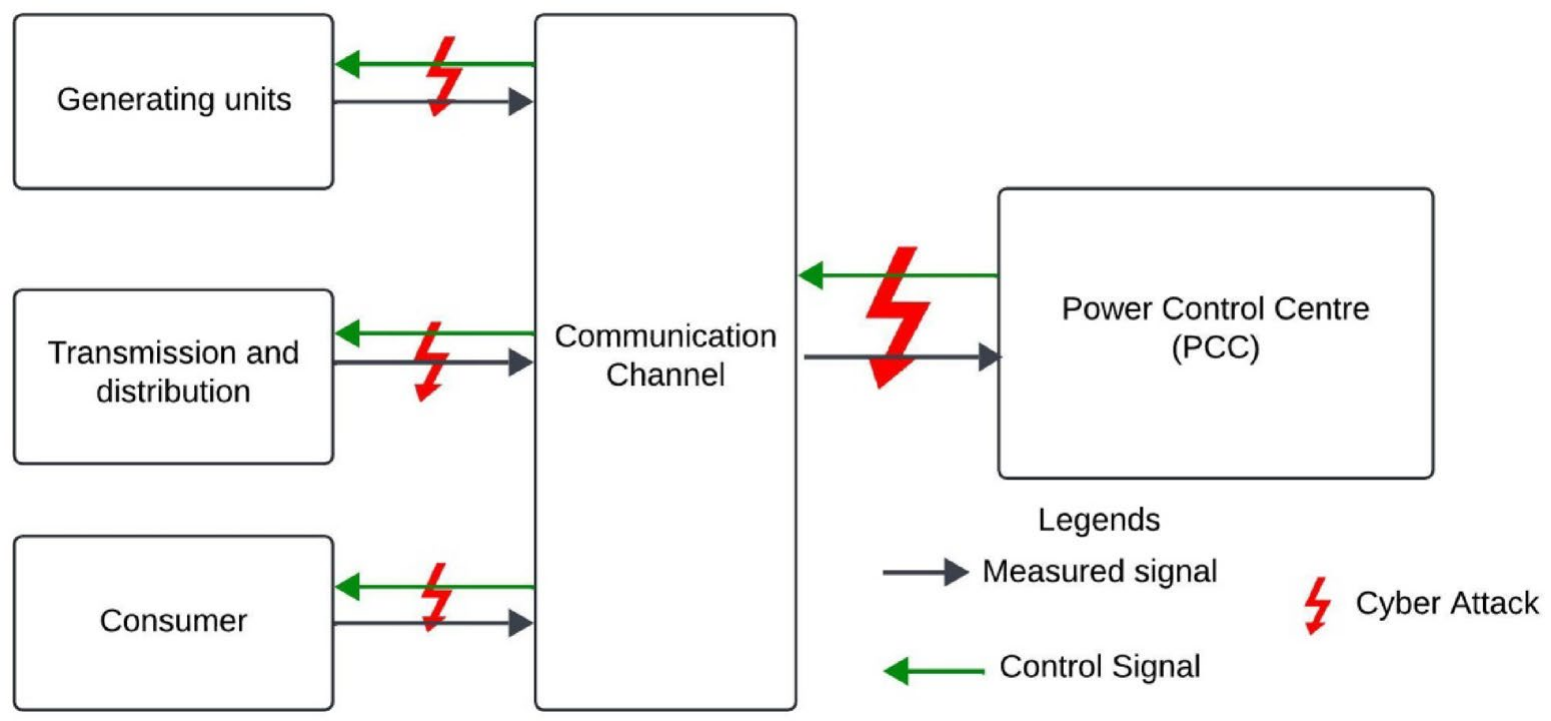
Possible cyber attack in communication channel of VPP.

As energy consumption continues to rise, traditional thermal power plants are increasingly unable to meet the growing demand, necessitating the integration of small-scale distributed generation systems at the consumer level to maintain the supply-demand balance. The Internet of Energy (IoE) plays a crucial role in enabling interaction between the energy market and consumers within the existing power grid. Alongside decentralized energy sources, end-user devices—known as prosumers—such as smart meters, smart appliances, smart inverters, and controllable loads, can now participate in the energy market (EM) through advanced information and communication technologies (ICT). The Virtual Power Plant provides a key framework for integrating renewable energy sources and end users into the energy market. As a software-based power plant, the VPP allows participants to connect in two ways: through an internet gateway, facilitating seamless communication and coordination^1^. The Virtual Power Plant enhances the monitoring and management of RES, enabling the efficient use of inverter-based generation capabilities. A central control system oversees the operation of all units within the VPP, processing crucial information such as bid quantities, bid prices, weather forecasts, and grid data. However, this complexity makes the system highly susceptible to cyber-attacks. The VPP’s ICT infrastructure is intricate, and its interoperability introduces several challenges, including resource allocation across different sources, secure communication, power scheduling, and overall system control. These issues must be addressed to ensure the system’s security and reliability^2^. The basic operation of a Virtual Power Plant and its potential vulnerabilities to cyber-attacks are illustrated in Fig. 1. The VPP can include a combination of conventional thermal power stations and small-scale renewable energy sources.

The system measures power flow data, while control signals are generated by the power control center (PCC) to manage the power system’s operations. To ensure the secure and reliable operation of the energy market, various standard protocols and architectural frameworks have been developed. Different communication architectures necessary for the VPP’s operation have been analyzed to address these security concerns^3^, it has been found that architectures based on the Common Information Model (CIM) and IEC 61,850 can effectively meet the communication requirements of VPP systems. However, the communication layer remains highly vulnerable to cyber-attacks. In addition to power system data, VPPs also handle sensitive system data, which attackers may exploit by targeting vulnerabilities within the infrastructure. Some types of attacks may go undetected in the early stages, highlighting the need for further research and advanced detection techniques. This study focuses on False Data Injection Attacks (FDIA), a prominent network-based attack targeting VPP systems. FDIA manipulates critical operational data, such as: altering energy market bids to generate financial losses or disrupt market operations and falsifying energy production or consumption data to mislead market participants or destabilize the grid. To address these risks, an unsupervised Autoencoder deep learning approach is proposed for detecting False Data Injection Attacks in VPP systems. AEs are particularly effective for anomaly detection because they learn intricate patterns from normal operational data and can recognize subtle deviations that may indicate cyber-attacks. By compressing input data into a lower-dimensional representation and then reconstructing it, the AE minimizes reconstruction error for normal data. However, when malicious data is introduced, the reconstruction error increases, signaling potential anomalies. This unsupervised approach eliminates the need for labeled attack data, making it highly suitable for dynamic and evolving VPP environments where attack patterns are constantly changing. The proposed AE model enhances the cyber security of VPPs by providing an automated, scalable, and adaptive detection mechanism that can identify attacks in real time, thereby ensuring the reliability and security of energy management systems.

### Related work

To address security concerns, researchers have proposed various strategies for detecting cyber intrusions and conducted impact analyses in the context of the smart grid and energy market. Paper^4^ highlights the security challenges in smart grids, emphasizing the importance of the confidentiality, integrity, and availability (CIA) triad as the primary objectives. A concise overview of common cyber-attacks, including DoS, spoofing, malware injection, and replay attacks, along with potential solutions, is provided in^5] and [6^. These attacks disrupt system objectives and interoperability. Paper^7^ discusses emulator-based cyber-physical analysis, showcasing the modeling and emulation of both communication and power systems.

The architecture, applications, impacts, and solutions for detecting cyber-physical security threats in the smart grid are examined in^8] and [9^. Specifically^8^, describes the deployment of the cyber-physical test-bed, which integrates industrial SCADA hardware and software with modeling and emulation techniques to establish a robust cyber infrastructure for the electric grid. Paper^9^ introduces a tool for detecting physical-layer cyber-attacks on the grid, employing an intrusion detection system (IDS) to identify and protect against known threats, such as Trojans, targeting the power grid. For any power system, when a malicious command is detected in real time, the operator can act fast to defend the whole system against these activity, averting cascading failures and ultimately blackouts.

The integration of electric vehicles into the smart grid’s cyber-physical system raises additional security and privacy concerns, as explored in^10^, which proposes a blockchain-based and software defined networking (SDN) detection technique to mitigate cyber threats in EV-connected smart grids. The aim of this work is to provide Protection of privacy and energy security to smart grid. Paper^11^ provides a brief review of IoT layers in VPP infrastructure, highlighting trending cyber-attacks and vulnerabilities. VPPs, being critical communication infrastructure, are particularly susceptible to attacks, as discussed in^12] and [13^. The National Electric Sector Cybersecurity Organization Resource (NESCOR) has identified various risks in the energy market, particularly for VPPs, as detailed in^14^. NESCOR has also outlined Critical Infrastructure Protection (CIP) standards, threat samples, evaluation methods, and safety criteria to ensure the secure and reliable supply of electrical energy to consumers. A framework for identifying the typical operating region of a VPP for cyber-secure operation is detailed in^15^. This work introduces cyber security analytics to define standard operating systems, using the typical operating region as a validation mechanism to detect malicious set-point requests in the network’s cyber layer. A denial-of-service (DoS) attack scenario involving a PV plant integrated into the power system is proposed in^16^, focusing on tie-line control actions as a primary attack point. This work employs a cellular convolution neural network for real-time detection of cyber-attacks. Attackers can also target power flow control from distributed generators during aggregation in the energy market, with the risk of jamming attacks in these operations discussed in^17^. An IDS based on Bayesian network graph for a power system is proposed in^18^. The process for building such a Bayesian network and determining the temporal-state changeover trends for various system scenarios is demonstrated in this research. These patterns are employed by the IDS as guidelines for categorizing comprehensible system situations and identifying intrusions that seek to compromise the security framework.

Paper^19^ explores how a DoS attack can penetrate the communication architecture of a VPP. It identifies the VPP’s voltage support and introduces a vulnerability index to quantify the impact of DoS attacks on demand response (DR) aggregation within the EM. Author in^20^ has proposed supervised, semi-supervised and online algorithms for detection of FDIA in smart grid and investigated the algorithms with various IEEE test systems and suggested attack detection framework, machine learning algorithms outperform attack detection approaches that use state vector estimation techniques in detecting attacks. Several forms of Support Vector Machines (SVM) and Naïve Bayes approaches have been tested for masquerade detection in the smart grid environment in^21^. A recent study^22^, presented Artificial neural network (ANN) based methods for controlling and monitoring of very common attack on VPP, the poisoning attack.

For VPP systems, FDIA detection and alarming system is proposed in^23^. A distributed economic scheduling of VPP units that takes security vulnerabilities into account is presented in this paper. FDIA considered as very potential cyber-attack in electricity market^24^. The concept of FDIA in power system introduce with the development of smart grid^25^ and the researchers are being attracted to work and find the novel solution for detection and mitigation of FDIA in smart grids^26–31^. In this study^27^, the authors mathematically define false data injection attacks with incomplete information from both the attacker’s and the grid operator’s perspectives. Additionally, they propose a novel vulnerability measure to compare and rank different power grid topologies based on their susceptibility to such attacks. An optimized clustering algorithm is introduced in^28^, considering the power system’s topology to classify potentially vulnerable nodes into different groups. Building on this, a state forecasting method is proposed to predict system states and detect FDIA. To evaluate the effectiveness and performance of the proposed approach, simulations are conducted on the IEEE 39-bus and IEEE 118-bus systems. Author in^29^ integrated k-means + + algorithm with expectation maximization algorithm and able to locate FDIA with probability of 95% for IEEE-5 bus and IEEE-14 bus smart grid systems. Drawing inspiration from federated learning, the study^30^ proposes an FDIA detection method based on secure federated deep learning, integrating Transformer, federated learning, and the Paillier cryptosystem. The study^31^, proposes an algorithm which combines CNN with sparrow search algorithm for localization of FDIA in smart grid and achieved high accuracy of 99.85% in localization with reduced false alarm only 0.03%. Another recent work^32^, explores cyber-attack identification using AI-based models namely decision trees and k-nearest neighbor (KNN), by estimating the state vector of the electricity network. Simulations are conducted using data extracted from the IEEE 5-bus network to evaluate the effectiveness of the proposed algorithm. False data attack vectors are injected into accurate measurements, and 2,000 measurement samples are collected—half representing healthy data and the other half manipulated data. After labeling the data accordingly, the proposed algorithms are applied to detect and classify attacks. A comparative analysis of the proposed algorithms against conventional methods reveals significantly improved accuracy. A comprehensive review on FDIA in smart grid system is presented in^33^ with detailed classification, challenges and its counterparts.

Paper^34^ presents analytics using a stacked autoencoder method to secure the transactive energy system. Two distinct rules for component outage and network-based attack detection have been proposed in this work. The suggested methodology detected 96.9% of attacks and 79% of component outages. The significance of IEC 61,850 in power system operations and the role of artificial intelligence in intrusion detection are thoroughly discussed in^35] and [36^. In^35^, the focus is on securing the smart grid’s communication layer (IEC 62351), where AI is employed to detect intrusions by analyzing the sampled values messages of IEC 61,850. This study achieved a high accuracy of 94.69% in minimal time using Extremely Randomized Trees (XRT) when the power system experienced both False Data Injection Attacks and faults. On the other hand^36^, explores the use of IEC 61,850’s Generic Object-Oriented Substation Event (GOOSE) signals to develop an intrusion detection system based on various machine learning techniques. Among the proposed methods, the Random Forest (RF) algorithm demonstrated the highest accuracy, achieving 95.19%, making it the most effective technique for identifying cyber threats in power systems. A critical review on the anomaly detection for various safety-critical systems has been presented in^37^. This study provide details about unsupervised ML method namely, Autoencoder and its uses in anomaly detection has been discussed.

In^38^, a cyber-secured economic dispatch model is proposed, addressing both colluding and non-colluding cyber-attacks. Malicious set-points can be created by attackers manipulating VPP cyber-layer settings. A more recent study^39^ offers a comprehensive review of potential cyber threats in the energy market, including cyber terrorism. This work emphasizes the critical need for thorough analysis in the energy sector, where control and command operations occur within interconnected environments, and highlight the challenges of monitoring, managing, and mitigating cyber security threats.

This literature review highlights various studies on detecting cyber-attacks in energy markets and smart grids. However, only a limited number of studies focus on VPP systems, which require further analysis to address different cyber-attack scenarios and detection methodologies to enhance cyber security in VPP environments. To tackle this gap, this work proposes an unsupervised Autoencoder machine learning (ML) strategy for detecting FDIA, which is mainly a network-based attack in the VPP environment.

### Contribution

The following are main contribution of this work:

i. To perform and detect FDIA in VPP systems, a 9-bus and IEEE-39 bus system model is proposed. This model includes four conventional power plant units, a solar photovoltaic (PV) system, energy storage devices, and controllable loads. The integration of these components enables a realistic representation of a VPP, capturing its complexity and operational dynamics. This framework facilitates the identification and evaluation of potential cyber-attack scenarios and their impact on the system.
ii. An unsupervised Autoencoder machine learning method is proposed to detect cyber-attacks. This approach leverages the Autoencoder’s ability to learn data patterns, enabling effective identification of anomalies and potential threats without requiring labeled datasets, making it well-suited for enhancing cyber security in complex and dynamic environments.
iii. This research aims to generate attack scenarios resembling normal operating data, including unit outages and electricity price manipulation, to analyze attack detection and assess their impact on the system’s normal operation. To validate the proposed model, the base system with integrated Virtual Power Plant units is utilized. This framework enables a comprehensive examination of both attack detection capabilities and their implications for normal operations, ensuring the robustness and reliability of the system in managing cyber security challenges.

To analyze FDIA in VPP, two key datasets are utilized: electricity price and output power from generating units. Specifically, the grid power output and PV system output are considered, with all data categorized into three groups: normal data, generating unit outages, and cyber-attack data. The remainder of the paper is organized as follows: Section “Security threats in VPP” presents a summary of security threats in VPP systems. Section “Methodology” provides a detailed explanation of the AE strategy. Section “Case ananlysis” investigates the proposed technique using a 9-bus VPP system, including a comprehensive discussion, followed by the conclusion of the work in Section “Conclusions”.

## Security threats in VPP

The proposed VPP system includes Conventional Power Plants (CPP), photovoltaic systems, energy storage systems, and controllable loads, all of which participate in the energy market. Ensuring cyber security in the EM is a significant challenge due to its vast scale and complexity, encompassing financial transactions, energy supply-demand dynamics, and other critical issues. The primary business objectives of an EM are secure interaction among components, secure monitoring and control, and Distributed Energy Resources operations^40^. Achieving these objectives requires fundamental cyber security measures, including maintaining the system’s safety, reliability, and resilience.

A typical IoT-based energy market system consists of three layers: the perception layer, network layer, and application layer. These layers communicate via a highly secure system, but the devices within them are vulnerable to security breaches. In VPP systems, these layers include devices such as sensors, SCADA, and Remote Terminal Units (RTUs) in the perception layer; communication and control devices in the networking layer; and smart meters, smart appliances, and Renewable Energy Systems in the application layer. The application layer also facilitates bidding prices and quantities, which are critical for EM operations, necessitating robust security measures^41^. Security threats in VPP systems may involve bid manipulation, market attacks, and changes to bid prices or Local Marginal Prices (LMP), or even attacks on substations. For instance, in December 2015, a cyber-attack on Ukraine’s power grid affected 30 substations. This was the first successful attack on a power grid, executed through spear-phishing emails that compromised the existing SCADA system^42^. Based on these insights, the potential attacks on VPP utilities can be categorized as follows:

*Attack based on the purpose*: The purpose of a cyber-attack may be to gain unauthorized access, slow down, or even crash the system. Access attacks, such as Man-in-the-Middle (MITM) attacks, target data interception and manipulation; while attacks aimed at slowing the system, such as Denial-of-Service (DoS) attacks, render the system inaccessible by overwhelming it with excessive requests. In^19^, the impact of DoS attacks on voltage support is analysed. Such attacks can disrupt the energy market by altering bid prices or quantities, flooding the system with high-volume data, and preventing VPP units from participating in bids. In severe cases, these disruptions can lead to system outages. MITM attacks occur when a hacker intercepts and manipulates communication between the load and the market server, positioning themselves between Distributed Energy Resources and the energy market. In this scenario, DER participants unknowingly interact with the attacker instead of the actual control server. This can result in energy theft and, in extreme cases, cause blackouts in affected regions.

*Attack based on the devices*: Anomaly injection or malware attacks can target the central control of the energy market (EM), leading to the destruction or leakage of sensitive information. Intrusions may also focus on physically tampering with grid equipment, protective devices, or prosumers, such as smart appliances. The cyber-attack in Ukraine serves as an example of malware injection at a substation. Common types of malware attacks include viruses, worms, ransomware, botnets, and keyloggers, among others. These attacks can compromise system integrity, steal data, or disrupt operations, posing significant risks to the security of the energy infrastructure.

*Attack based on the network*: The operation of a Virtual Power Plant heavily relies on its information and communication technology (ICT) infrastructure. Attacks targeting the application, transport, and network layers, as well as multi-layered attacks, can prevent servers from detecting and transmitting critical information across the network. As a result, attackers may manipulate vital energy market data, such as bid quantities or bid prices from prosumers, and alter the settings of smart appliances, including temperature, frequency, and voltage. These attacks aim to illegally modify or distort data within the VPP. False Data Injection Attacks (FDIA) is most popular attack in the network based type^26,40,41^. False Data Injection Attacks target the network, attempting to corrupt readings from power grid sensors and phasor measurement units, thereby misdirecting the operation and control centers. Such attacks can cause unavailability of information for VPP units, preventing their participation in the market, or they may alter the Local Marginal Price of electricity, ultimately impacting electricity pricing. FDIA can be classifying in two categories depending on where the false data is being injected namely: (i) False energy data injection and, (ii) false communication link-state data injection. Furthermore, both supply and demand nodes are susceptible to injecting fake energy data; hence both nodes must be taken into account for security reasons. In order to create a false data state, an intruder can also target the communication channels utilized for data and energy transmission. This could result in higher transmission costs and an imbalance in the energy market’s power flow.

**Fig. 2:**
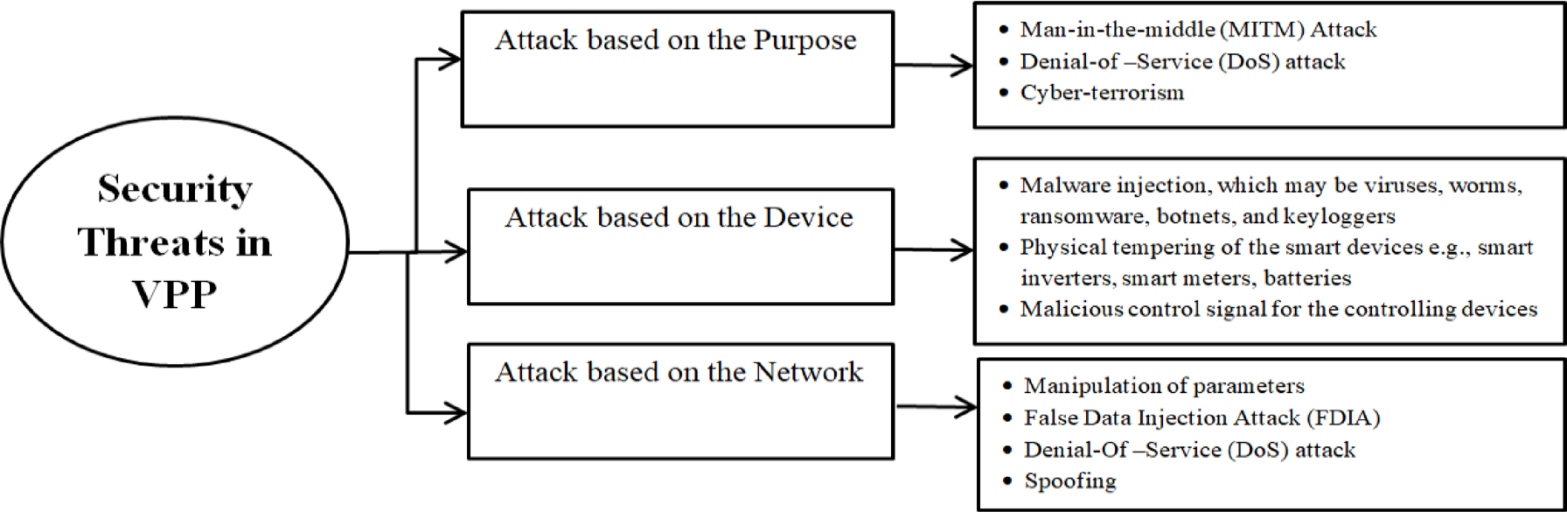
Possible Cyber-attack in VPP Systems.

The impact on the energy market and other VPP operations will vary depending on the severity of the cyber-attack. Therefore, it is crucial to analyze these impacts and develop detection and mitigation strategies to ensure a secure and safe energy market. Among the various cyber threats, network-based attacks specially, FDIAs are attracting significant attention due to their potential to affect not only the energy market but also finance, governance, and other critical areas and so, in this work False data injection attack has been chosen for the analysis. (Fig. 2).

## Methodology

Detecting the malware intrusion in VPP environment is very challenging issue as the component of the VPP and its participation in energy market is uncertain. ML techniques may be the ideal solution for this. Paper^43^ uses Naïve Bayes algorithm and J48 bagging tree model for investigating the economic risks due to cyber-attack in VPP system. Also, the effects of cyber threats and for providing the economic dispatch model of VPP has been proposed by neighborhood-watch based resilient energy management system^44^. AE and Convolution Autoencoder (CAE) based anomaly detection technique have been presented in^45^ for widely used NSL-DDL dataset. Also author shown the comparison between different methodologies for anomaly detection and the proposed techniques gives the better accuracy. The false positive rate and accuracy rate for AE in this study is 4.09% and 95.85%. In^46^, author proposes an AE technique for network based anomaly detection for cloud networks with CIDDS-001 dataset, where the reconstruction error is considered as anomaly metric. As highlighted in^46^, the proposed Autoencoder (AE)-based method achieved an impressive 100% accuracy with a 0% false positive rate, making it an intriguing prospect for researchers. In studies^47] and [48^, AE approaches were applied to solar power forecasting and time-series forecasting, respectively, demonstrating their versatility in predictive analytics. The use of AE models for anomaly detection is not confined to power systems or computing applications; they have also been successfully implemented in the transportation sector^49^ and manufacturing processes^50^. In^49^, two distinct AE-based models were developed to assess risks associated with the increasing autonomy of vehicles. Similarly, in^50^, AE techniques were explored to address defect recognition challenges without requiring defect samples for training. Among various supervised and unsupervised machine learning (ML) techniques, AEs are increasingly being recognized as a preferred choice for cyber intrusion detection and time-series analysis. Their effectiveness stems from their ability to capture complex patterns in data and identify anomalies with high precision. Autoencoders, as a type of neural network, offer several advantages over traditional ML methods, making them a valuable tool in diverse fields. The key benefits of using AEs for anomaly detection can be summarized as follows:

- Dimensionality Reduction: AEs excel at reducing high-dimensional data to a lower-dimensional latent space while preserving critical information. This makes them ideal for processing complex datasets with many features, which is often the case in cyber security and time series data.
- Handling High-Dimensional and Imbalanced Data: AEs are well-suited for datasets that are high-dimensional and contain class imbalances, such as datasets with rare events or anomalies. They can effectively identify patterns in the majority class while highlighting deviations that indicate anomalies.
- Feature Extraction: AEs automatically learn and extract meaningful features from raw data during training, eliminating the need for manual feature engineering. This ability enhances their effectiveness in identifying complex, hidden patterns associated with anomalies.
- Noise Reduction: AEs have the capability to denoise data, making them valuable for processing noisy datasets. By learning a compressed representation of the data, AEs can reconstruct clean versions of the input, aiding in more accurate anomaly detection.
- Ease of Understanding and Application: Despite their neural network foundation, AEs are relatively straightforward to understand and implement. Their architecture and functionality can be easily adapted for a wide range of anomaly detection applications.
- Scalability: AEs are highly scalable, making them suitable for large-scale datasets commonly encountered in cyber security and time series analysis. Their ability to process large volumes of data efficiently is a key advantage for modern applications.

These attributes make Autoencoders a powerful and versatile tool in the realm of anomaly detection and analysis, particularly in domains like cyber security and time series forecasting, where detecting subtle anomalies is critical.

**Fig. 3:**
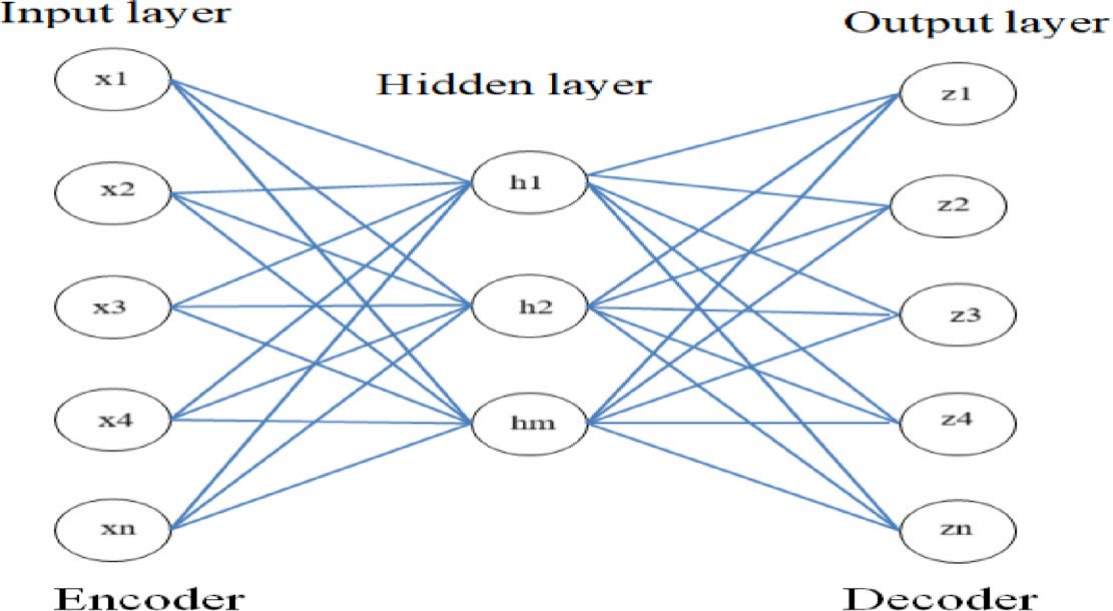
The Autoencoder architecture.

AE is an unsupervised deep learning method, consisting of three layers- encoder (input layer), multiple hidden layer and decoder (output layer), shown in Fig. 3. The function of encoder is to map the input data into hidden layer (AE layer) with lower dimension from the input dimension. Finally, decoder maps the hidden layer and reconstructs the original data with the same dimension as of input layer. So the training process in AE has two phases encoding and layer *z* can be represented as: decoding. For input data *x*, the hidden layer *h* and reconstructed output *z* can be represented as given in Eqs. (1) and (2):

1 $$h{\text{ }} = {\text{ }}\sigma \left( {{W_1}x{\text{ }} + {\text{ }}{b_1}} \right)\;$$

2 $$z{\text{ }} = {\text{ }}\sigma \left( {{W_2}x{\text{ }} + {\text{ }}{b_2}} \right)$$

Where, *σ* is activation function and *W*_*1*_, *W*_*2*_, *b*_*1*_, and *b*_*2*_, weight matrix and bias vectors for encoder and decoder respectively. In this work rectifier linear unit (ReLU), activation function has been used and is given by Eq. (3) for all layers except output layer,

3 $$\sigma \left( x \right){\text{ }} = {\text{ }}{x^ + }max\left( {0,x} \right)$$

There is the need of preventing from overfitting during training process and this can be achieved by randomly activation of each node based on Bernoulli distribution with probability p. then the output of any layer *i + 1* will be given by Eq. (4),

4 $${x_{i + 1}} = {\text{ }}\sigma \left( {{W_i}\left( {{r_i}*{\text{ }}{x_i}} \right){\text{ }} + {\text{ }}{b_i}} \right)$$

Where, *r*_*i*_ is the sampled value from Bernoulli distribution. The loss function is defined for reducing the reconstruction error between *x* and *z*.

Input dataset of AE is categorized into training, validation, and field deployment for various purposes. AE learnt connection weights based on training and validation data. During the training the hyperparameters are tuned including stopping points. After tuning the model, data is put into the AE to estimate its performance. To detect anomalies, only normal data is used for training and validation. The trained model is then evaluated on both normal and anomalous events.

To account for varying signal magnitudes, three groups of data are normalized using z-score normalization, i.e., the mean and standard deviation from training data. The z-score normalization is given by Eqs. (5),

5 $$\:\stackrel{\sim}{x}=\:\frac{x-{\stackrel{-}{x}}_{\text{m}\text{e}\text{a}\text{n}}}{{s}_{deviation}}$$

Where, $\:\stackrel{\sim}{x}$ is the normalized value of *x*, $\:{\stackrel{-}{x}}_{\text{m}\text{e}\text{a}\text{n}}$ and $\:{s}_{deviation}\:$are mean and standard deviation of the training data. During training, loss function should be minimized. To determine the optimal collection of hyperparameters, many models are trained and tested on a validation dataset. There is tuning of threshold for anomaly detection rules to ensure that the fraction of validation data with a reconstruction error below the threshold is met. AE model have low dimensional hidden layer with small reconstruction error. The attacks or anomalies are detected as per threshold set during the validation process. The reconstruction error, *r* is defined as the difference between the reconstructed output vector *z* and the input vector *x* and is given as,

6 $$\:r=\:\parallel x-z \parallel$$

**Fig. 4:**
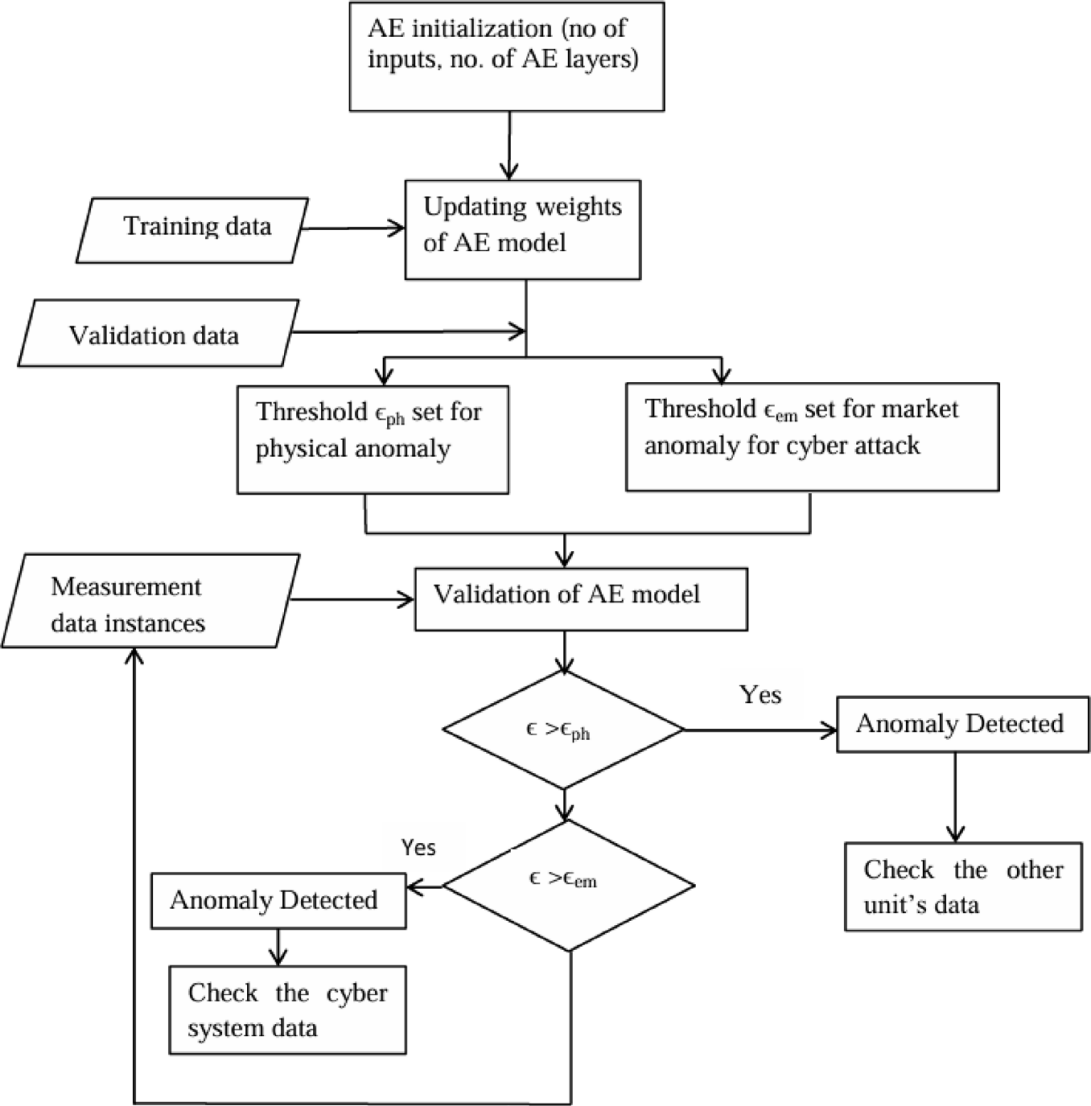
Flowchart of proposed model.

The aim of the AE is to reduce the value of *r.* The overall process the proposed model has been depicted in Fig. 4.

### Hyperparameter tuning

Hyperparameter tuning in an AE involves adjusting various model parameters to optimize performance. One effective way to guide this tuning process is by monitoring the reconstruction error, which measures how well AE can reproduce its input data.

Step for Hyperparameter Tuning with Reconstruction Error.

1. Define the Reconstruction Error: The reconstruction error is typically calculated using: Mean Squared Error (MSE), Mean Absolute Error (MAE) and Binary Cross-Entropy (BCE).
2. Select Hyperparameters to Tune: Key hyperparameters in an autoencoder include: Number of neurons, Learning Rate, Number of Layers & Neurons, Activation Functions, Dropout Rate, Batch Size, Epochs.
3. Train the Autoencoder: Train the model using training data and monitor the validation loss (reconstruction error on validation data).
4. Monitor Reconstruction Error: Track the error for both training and validation datasets. A high validation error may indicate: Overfitting (if training error is low but validation error is high) and Underfitting (if both training and validation errors are high).
5. Tune Hyperparameters Based on Reconstruction Error: check the reconstruction error whether this is too high or overfits the model or the training is going too slow. In these cases the number of neurons, number of layer and leraning rate play an effective role so these needs to keep updated according to the condition arises.
6. Use Grid Search or Bayesian Optimization or Random search to test the combinations of hyperparamerts.
7. Evaluate the Best Model: And the final step is to test the model on unseen data to confirm its generalization ability and Compare models based on reconstruction error distribution or anomaly detection performance (if used for anomaly detection).

The hyperparameters taken for this work has been summarized in Table 1.

**Table 1:** Hyperparameter for Ae model.

Parameters	Values
Activation Function	ReLU
Loss Function	MSE
Optimizer	Adam
No. of nodes in the hidden layer	5
Dropout	0.2
Shuffling	Every Epoch
Batch size	128
Epochs	120
Learning rate	0.001
No. of neurons	32
No. of Convolutional layer present	5
No. of filters	16
Threshold	1.1 *r*

### Evaluation metrics

In time series data analysis, two types of errors are typically assessed to evaluate detection accuracy and performance: scale-dependent errors and percentage errors^48,51^. Scale-dependent errors are measured on the same scale as the data, which limits their usefulness for comparing series with different scales. In this study, two well-known scale-dependent error metrics are used: mean absolute error (MAE), and root mean square error (RMSE) are given by Eqs. (7) and (8) respectively,

7 $$\:MAE=\:\frac{1}{n}\sum\:_{i=1}^{n}\left|{z}_{i}-\:\widehat{{z}_{i}}\right|$$

8 $$\:RMSE=\sqrt{\frac{1}{n}\sum\:_{i=1}^{n}{\left({z}_{i}-\:\widehat{{z}_{i}}\right)}^{2}}$$

Where, $\:{z}_{i}$ is the actual data and $\:\widehat{{z}_{i}}$ is the reconstructed output data from AE model. For higher accuracy of proposed system model, MAE, RMSE and MAPE should be lower.

## Case ananlysis

### Data generation

**Fig. 5:**
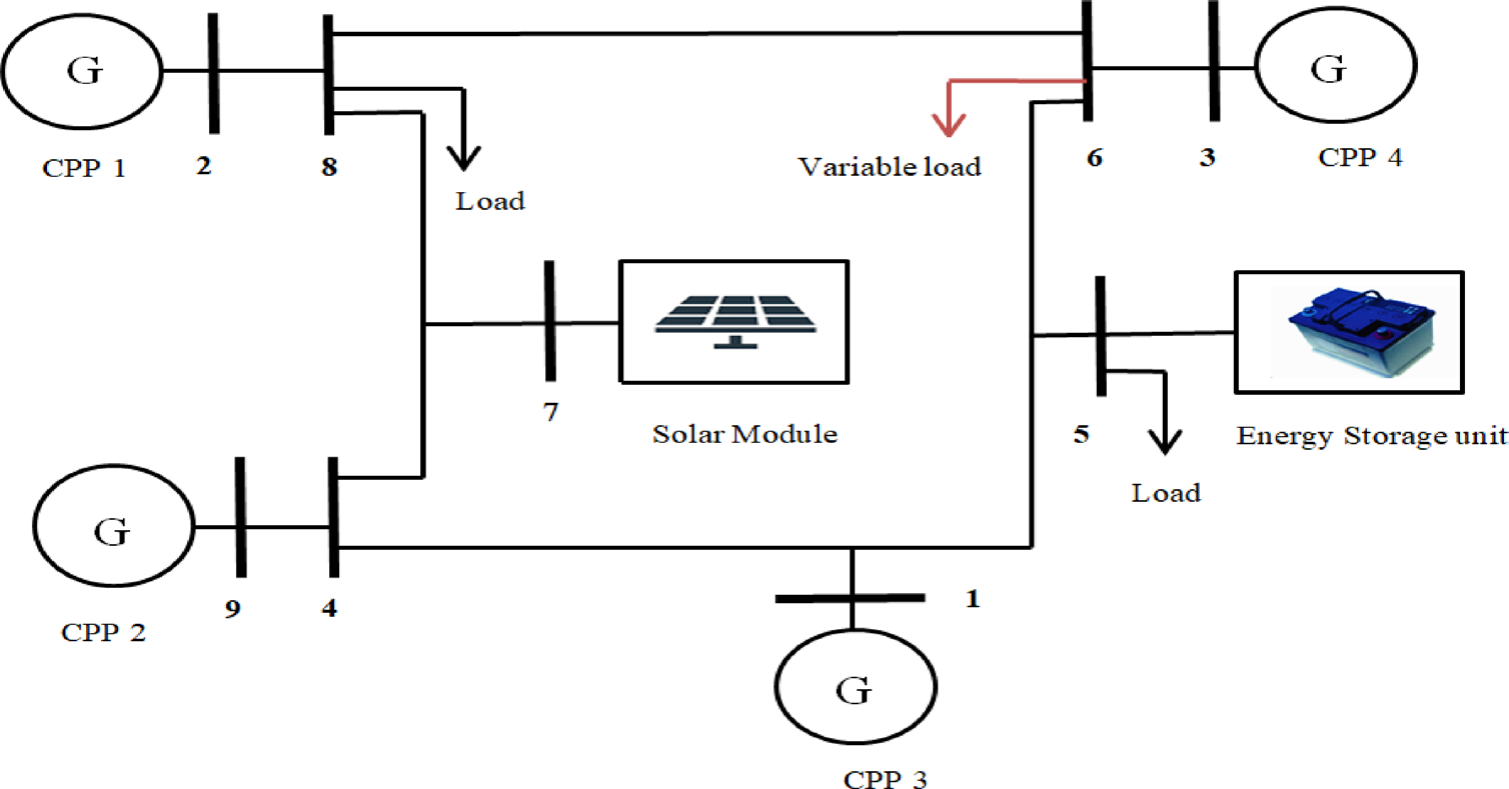
Modified 9-bus System structure.

**Fig. 6:**
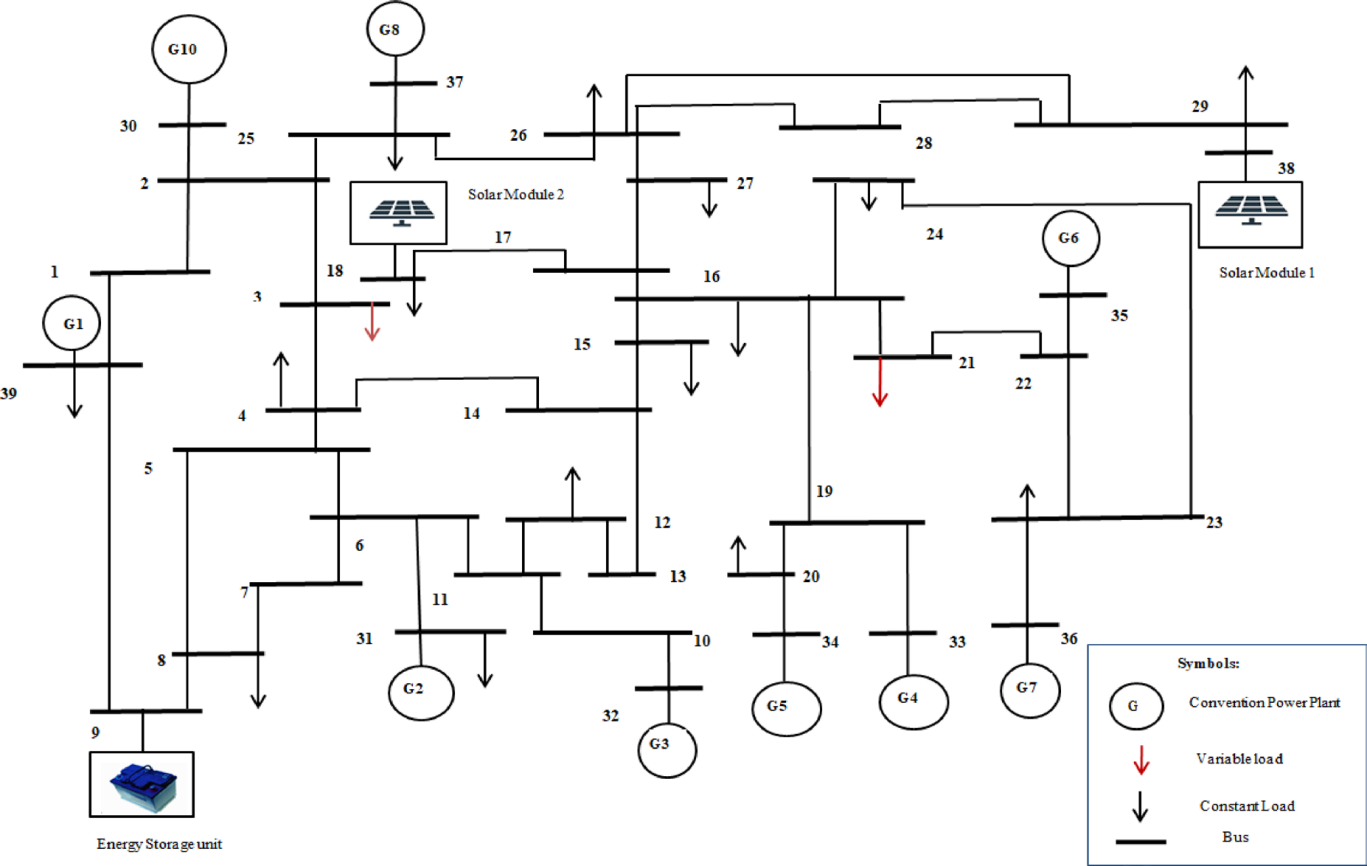
Modified IEEE-39 bus System structure.

The VPP system used for validating the proposed methodology is illustrated in Figs. 5 and 6. The system under consideration is a 9-bus distribution network and modified IEEE-39 bus system integrated with various Renewable Energy Sources. It is modelled and simulated in MATLAB Simulink to generate time-series data, which is subsequently utilized for FDIA detection using an Autoencoder. The simulated VPP system comprises the following components: Four conventional generating units, solar photovoltaic (PV) system, Energy storage system (ESS), Variable loads and constant load. In 9 bus system one unit of solar PV module with ESS system has been integrated at bus 7 and 5 respectively (Fig. 5). The modified IEEE-39 bus system has 9 units of CPP units, two solar PV modules, one ESS and two variable loads, which are connected at bus 38, 17, 9, 18 and 21 respectively (Fig. 6).

The output variables of the system include the power output from the grid, PV power, state of charge (SOC) of the ESS, load demand, and electricity price. Among these output variables, PV power and grid power are selected as bid quantities for participation in the energy market. The cyber-attack scenarios are designed around these bid quantities and the electricity price for consumers. Data collection for the VPP system was carried out over a period of 1000 days under three different operational scenarios: (i) Normal Operation (700 days): The system operates under standard conditions without any faults or attacks, (ii) Component Outage (200 days): During this period, one of the conventional generating units is assumed to be faulty and out for maintenance, and (iii) Manipulation on Electricity Price, 100 days. For these days, the electricity price is altered within a range of (0–30). The impact of these cyber-attacks is analysed in terms of changes in bid quantity data, outages of generating sources, and variations in electricity prices. These variations directly influence the system’s reliability and performance, highlighting the importance of robust detection mechanisms. The input data and system parameters used in this study are summarized in Table 2. This comprehensive dataset serves as a crucial foundation for analyzing the impact of cyber-attacks and validating the proposed FDIA detection methodology using AE. By encompassing a wide range of scenarios, it enables a thorough assessment of the model’s effectiveness under diverse operational conditions. In this study, component outages are not treated separately from attack conditions; instead, they are collectively considered as part of the overall attack criteria. However, future research can focus on distinguishing FDIA from generator outages more precisely, allowing for a more granular analysis. This distinction could enhance the model’s accuracy in identifying specific threats and improving grid resilience. Additionally, incorporating real-time data and expanding the dataset to include more diverse attack vectors and outage scenarios may further optimize detection capabilities. Future work could also explore hybrid approaches, combining AE with other deep learning techniques to achieve higher accuracy and robustness in detecting cyber-physical threats in power systems.

**Table 2:** Input data for Ae model.

Data	Size of Data
Training data	700 Days
Validation Data	200 days
Field deployment data	100 days

### Simulation, and results

The data generated from the MATLAB Simulink model has been utilized to validate the performance of the proposed AE model for anomaly detection in the VPP system. The methodology involves training the AE model with 700 days of normal operation data, validating it with 200 days of data representing a component outage scenario, and testing it with 100 days of attacked data to assess its real-world performance for field deployment. The AE model is trained exclusively on normal operating condition data. During this process, the model learns the hidden representations inherent in the typical system behavior, such as patterns and relationships between variables. The AE’s primary objective is to reconstruct the input data as accurately as possible. When properly trained, the AE generates output that closely matches the input for normal operation data. The reconstruction error, which is the difference between the input and output, is used to identify anomalies. If the reconstruction error exceeds a predefined threshold, it indicates a deviation from normal behavior, flagging the presence of an anomaly. This approach ensures that the AE model effectively distinguishes between normal and anomalous conditions.

The training phase includes the following key parameters: Number of epochs: 120 (an epoch refers to one complete pass of the training dataset through the model), Iterations per cycle: 7 and Maximum number of iterations: 840 (total iterations conducted during training). During the training phase, the AE model effectively captures prominent features within the input data by focusing on areas with frequent signal peaks and distinct patterns. This capability enables the model to comprehensively learn the system’s operational behaviour and accurately replicate it during the reconstruction phase. The reconstruction error, measured using key evaluation metrics namely: Mean Absolute Error (MAE) and Root Mean Square Error (RMSE), serves as the primary performance indicator of the AE model.

**Fig. 7:**
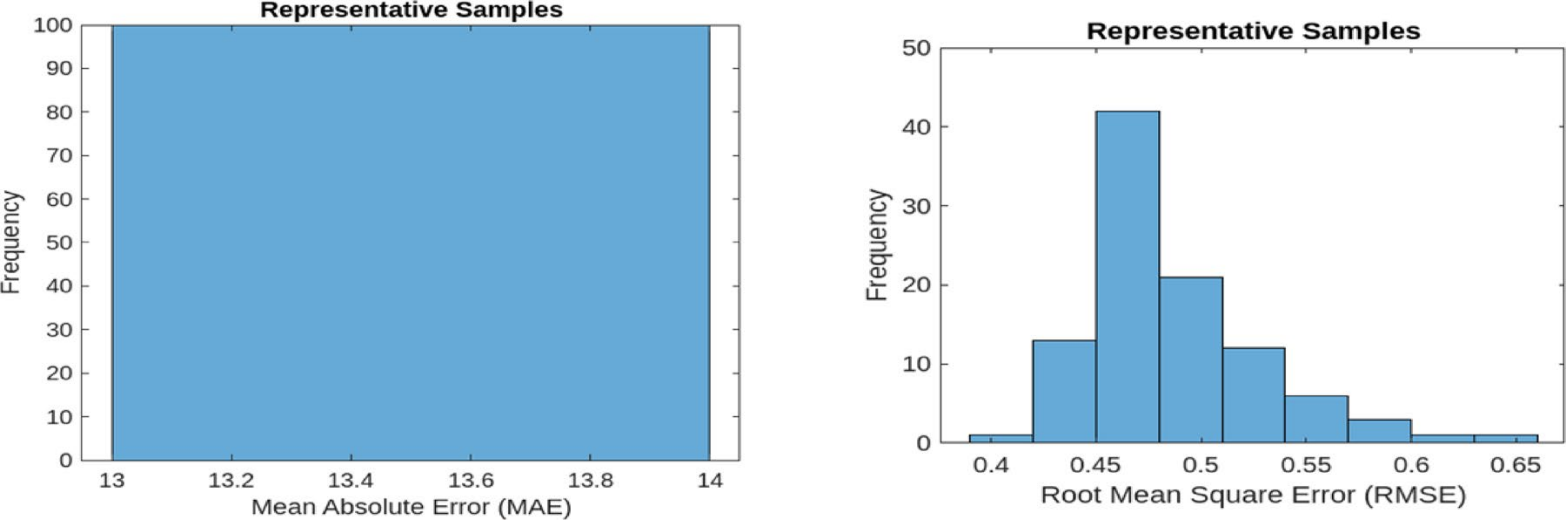
MAE and RMSE for Representative samples for 9-Bus System.

**Fig. 8:**
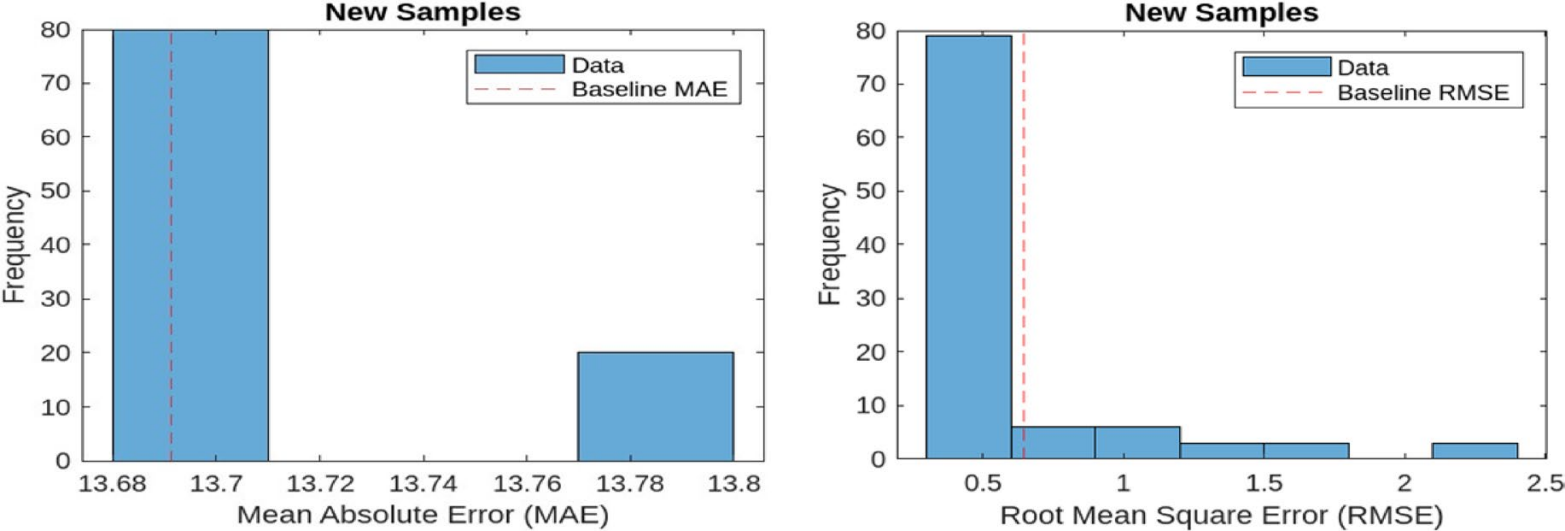
MAE and RMSE for New samples for 9-Bus System.

**Fig. 9:**
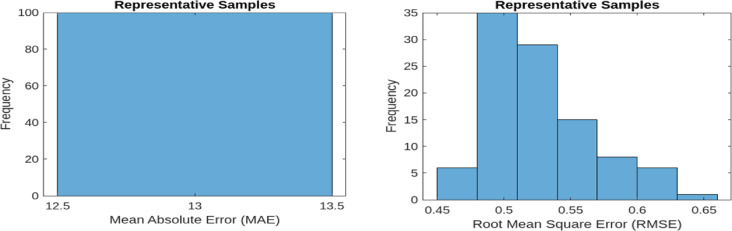
MAE and RMSE for Representative samples for IEEE-39 Bus System.

**Fig. 10:**
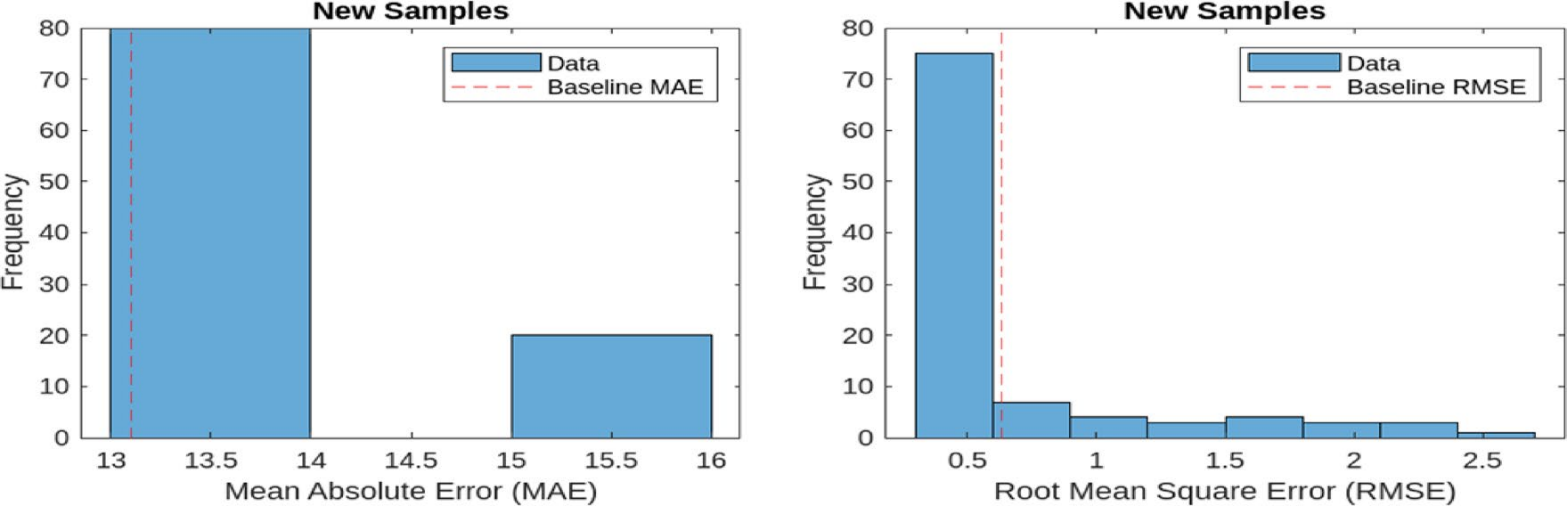
MAE and RMSE for New samples for IEEE-39 Bus System.

MAE quantifies the average of the absolute differences between the predicted and actual values. A lower MAE value signifies improved performance in reconstructing normal operational signals. The baseline MAE is recorded at 13.60 and 13.1044 for 9 bus and IEEE 39 bus respectively. RMSE, on the other hand, measures the square root of the mean squared differences between predicted and actual values. As RMSE is more sensitive to larger errors, it provides a stricter evaluation of reconstruction performance. The baseline RMSE for the model stands at 0.6041 and 0.6719 for 9 bus and IEEE 39 bus respectively. These evaluation metrics are applied to both representative samples of normal operational data and new anomalous data samples. Figures 7 and 8 illustrate the MAE and RMSE values for representative samples (normal samples) and new samples (anomalous samples) 9 bus system and so Figs. 9 and 10 is for IEEE 39 bus system which effectively showcasing the model’s capability in distinguishing anomalies by comparing reconstructed outputs against the original inputs.

**Fig. 11:**
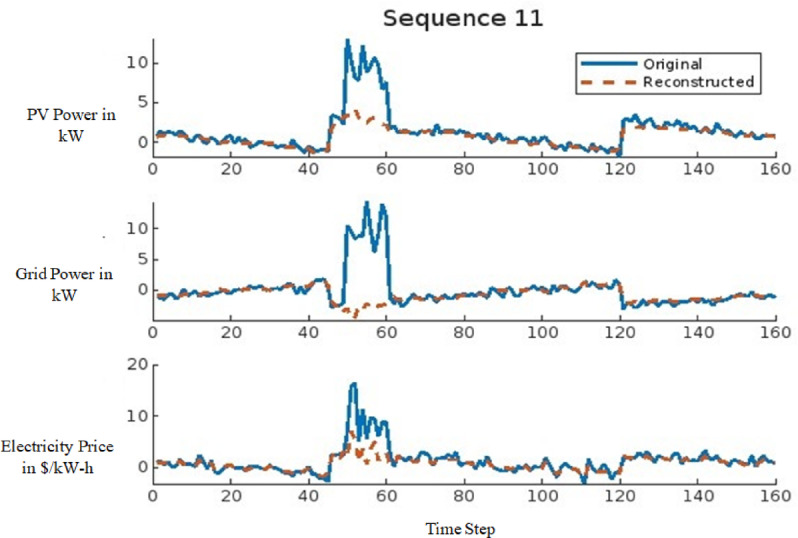
Sequence 11 showing the original and reconstructed signal for small data.

**Fig. 12:**
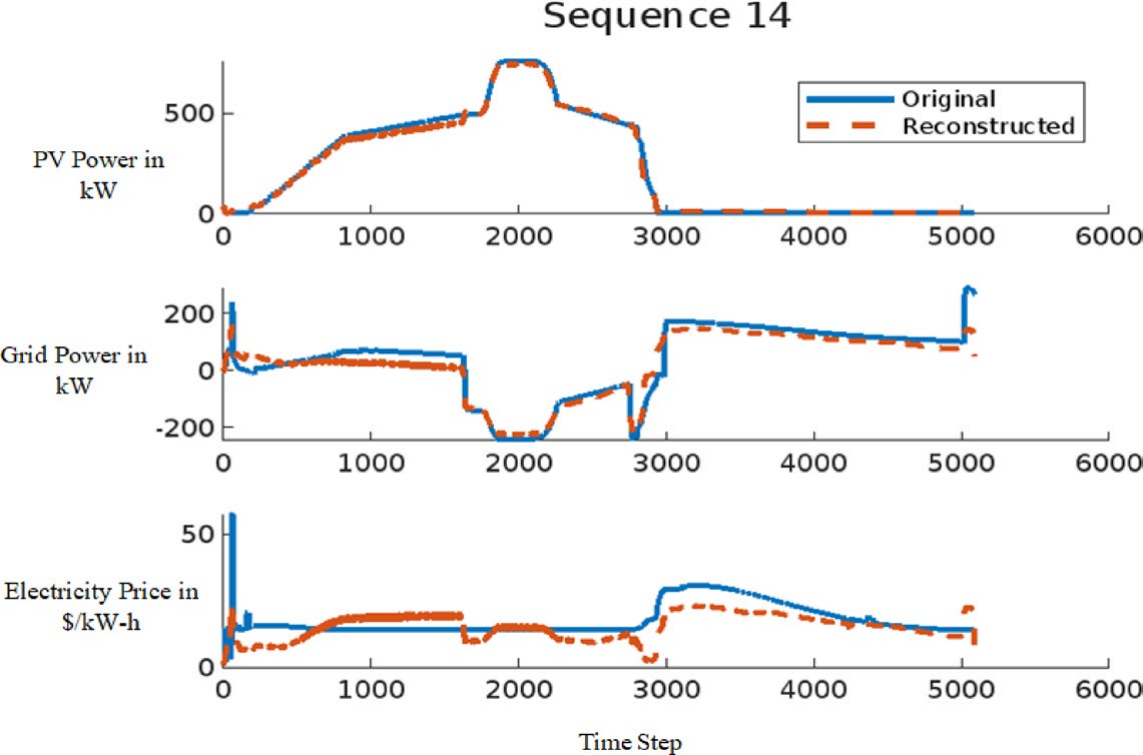
Sequence 14 showing the original and reconstructed signal for large data.

**Fig. 13:**
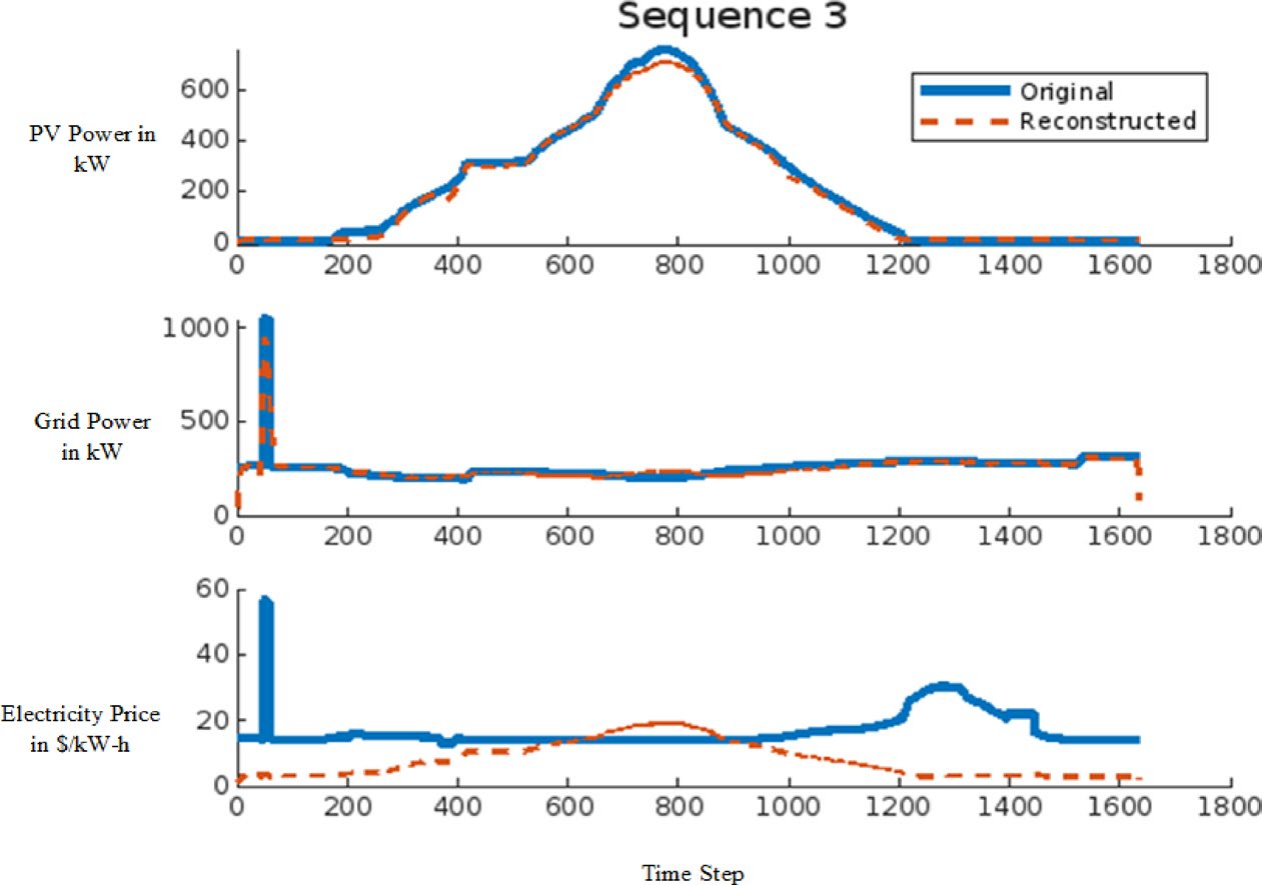
Sequence 3 showing the original and reconstructed signal for modified IEEE-39 bus System.

The signal reconstruction process represents the final stage of the AE model, where the model attempts to reconstruct the input signal from anomalous data. This stage is critical in assessing the model’s capability to differentiate between normal and anomalous data. The effectiveness of the AE model in reconstructing signals is demonstrated in Figs. 11 and 12 for a 9-bus system and in Fig. 13 for the IEEE 39-bus system. Figure 11 illustrates the comparison between the original and reconstructed signals for sequence 11, focusing on a dataset with small variations. In contrast, Fig. 12 presents sequence 14, highlighting large-scale data variations. Similarly, Fig. 13 showcases the original and reconstructed signals for sequence 3 within the proposed IEEE 39-bus system. These figures collectively indicate that the AE model can accurately reconstruct normal signals while identifying deviations when anomalies, such as FDIAs, are present. The primary objective of this study is to detect FDIA attacks using the AE model, where the reconstructed signal serves as an indicator of system integrity. By analyzing the differences between the original and reconstructed signals, the AE model can effectively identify and isolate anomalies, thereby enhancing the security and reliability of the power system.

**Fig. 14:**
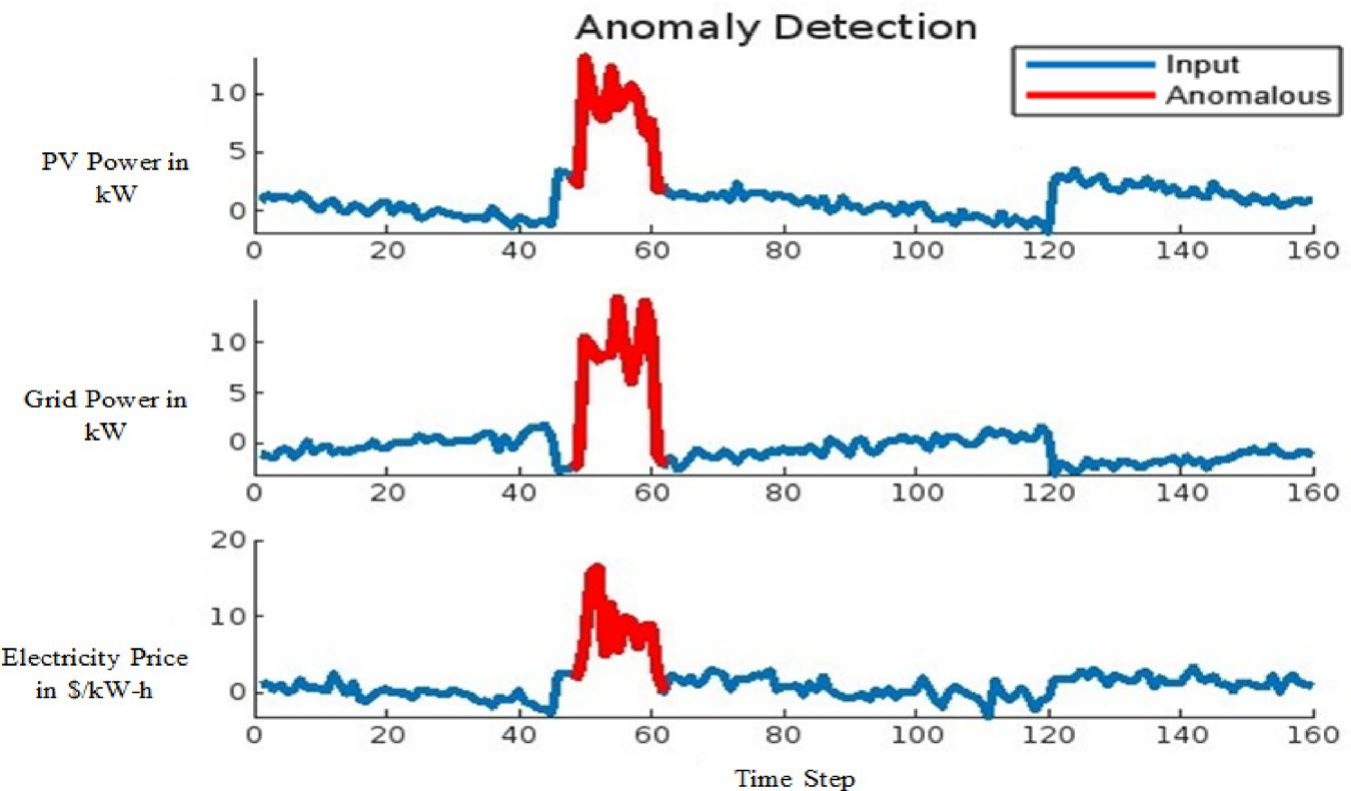
Anomaly detection for small data for 9-bus system.

**Fig. 15:**
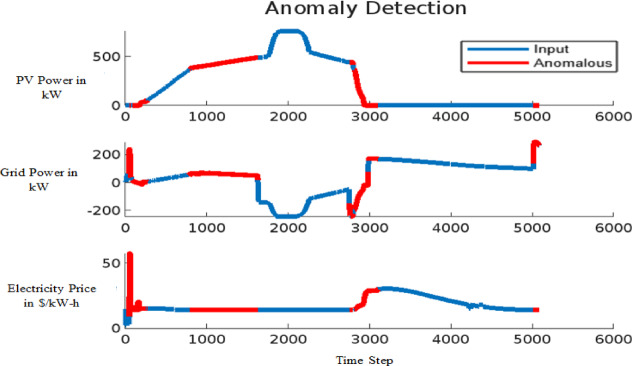
Anomaly detection for large data for 9-bus system.

**Fig. 16:**
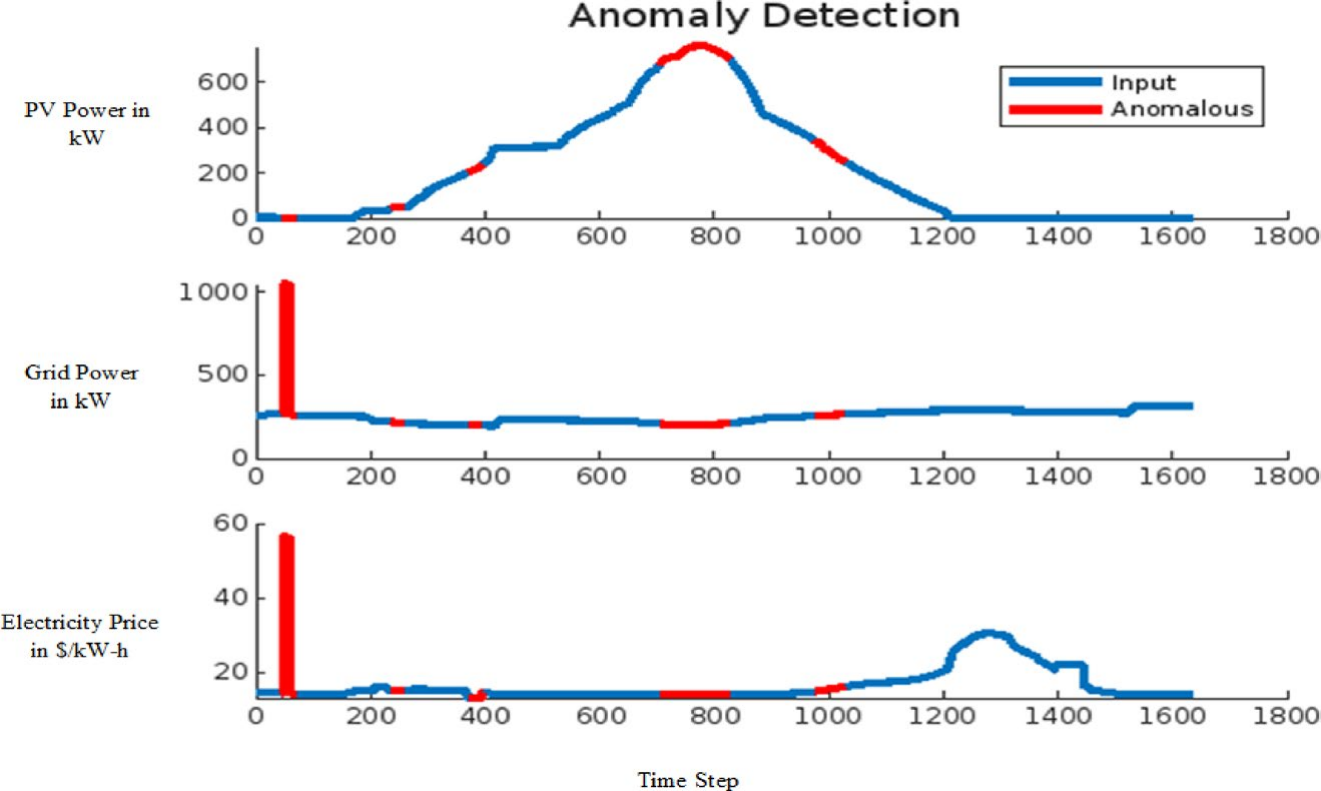
Anomaly detection for modified IEEE-39 Bus system.

The effectiveness of the model in detecting anomalies induced by FDIA is demonstrated in Figs. 14 and 15 for the 9-bus system and Fig. 16 for the IEEE 39-bus system. In these figures, anomalous data points are highlighted in red, showcasing deviations in critical system parameters such as conventional grid power output, solar PV power generation, and electricity prices within the VPP market. Figure 14 presents the anomaly detection results for small-scale datasets, while Fig. 15 offers insights into the detection of large-scale anomalies. In both cases, the red line distinctly marks anomalies, ensuring a clear differentiation between normal operational data and compromised data points. This visual distinction highlights the model’s capability to effectively identify and isolate anomalies, reinforcing its reliability in safeguarding power system operations against FDIA threats.

### Performance and discussion

**Fig. 17:**
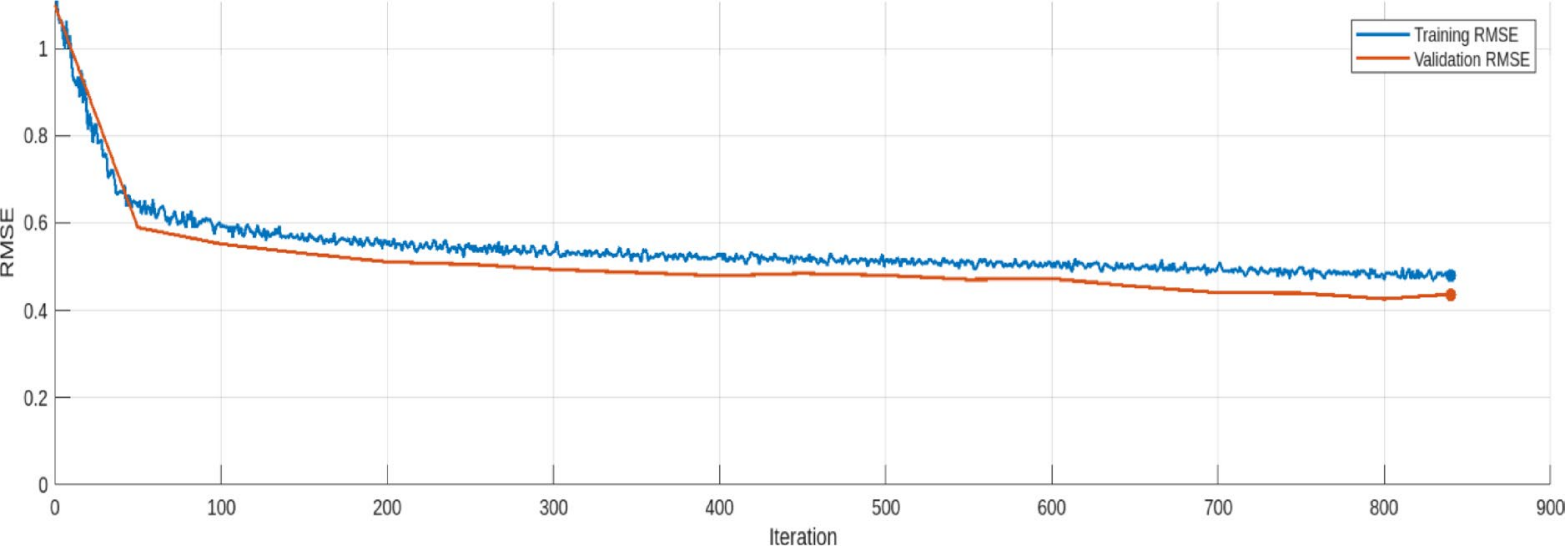
RSME during Training and Validation process 9-bus system.

**Fig. 18:**
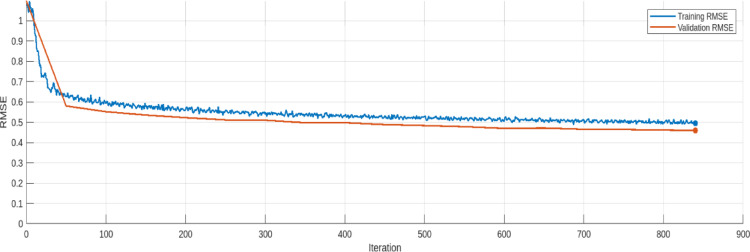
RSME during Training and Validation process modified IEEE 39-bus system.

**Fig. 19:**
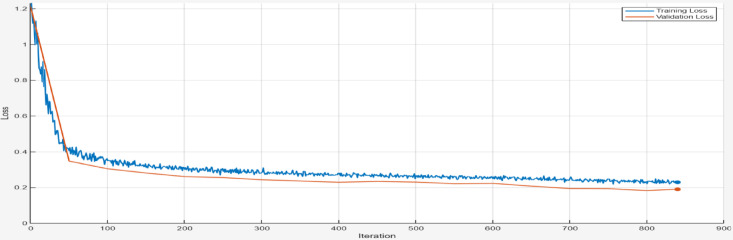
Loss during Training and Validation process for 9-bus system.

**Fig. 20:**
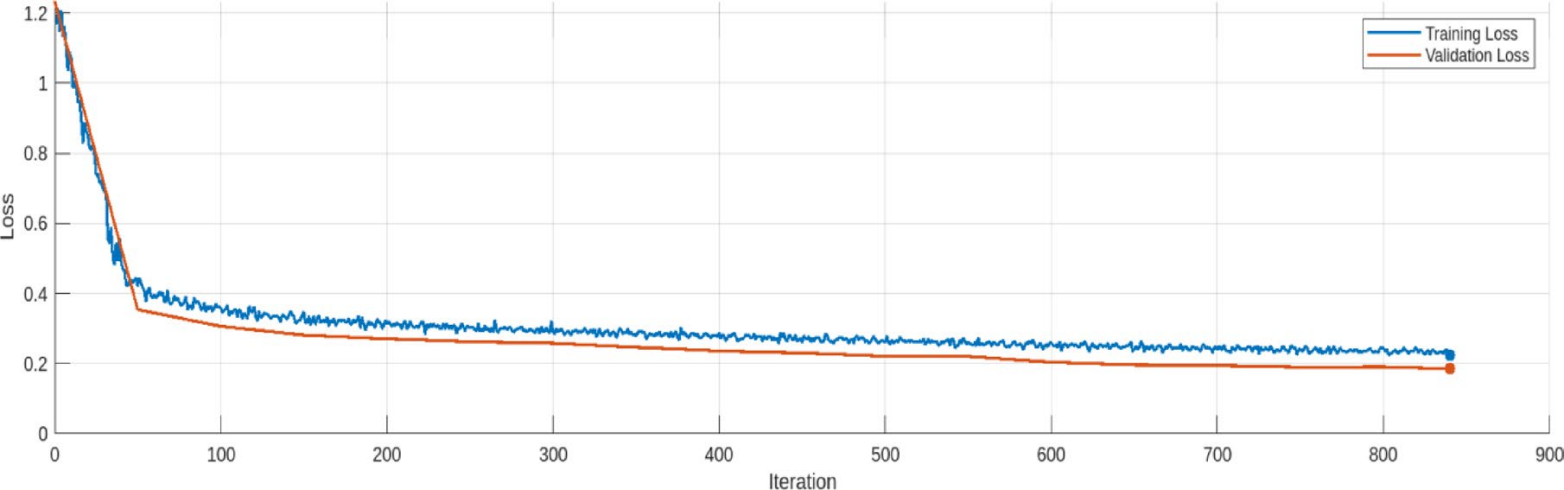
Loss during Training and Validation process for modified IEEE 39-bus system.

**Fig. 21:**
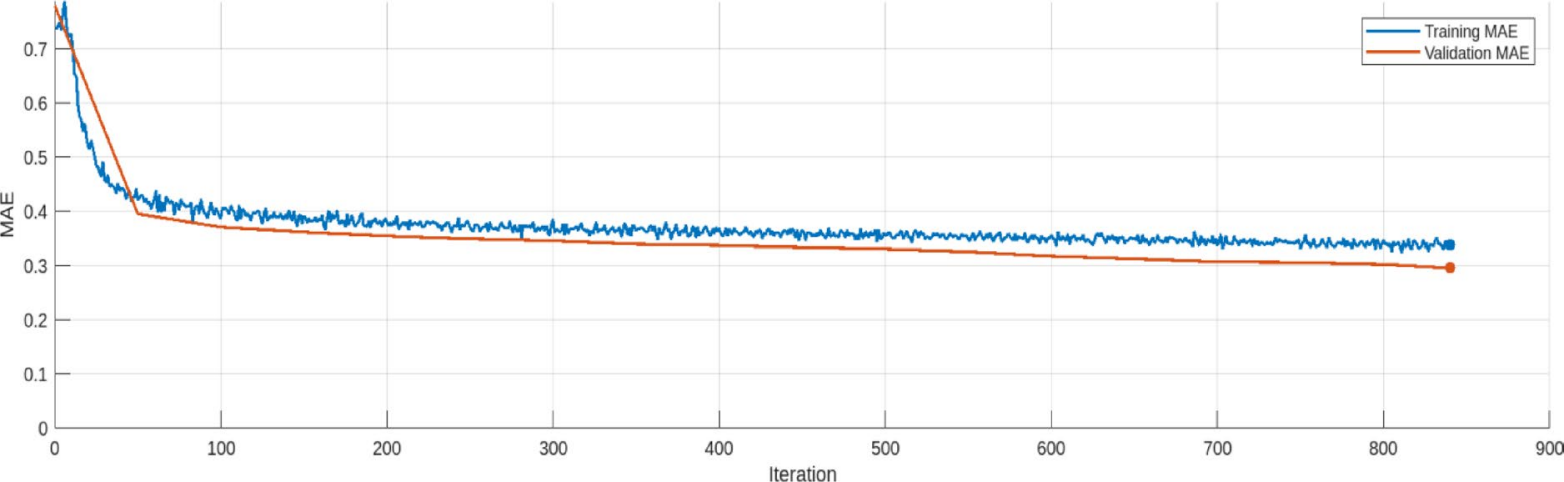
MAE for Training and Validation process for for 9-bus system.

**Fig. 22:**
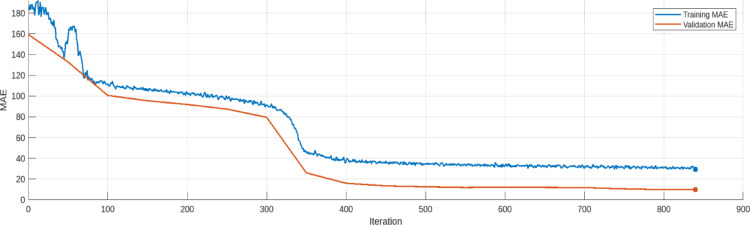
MAE for Training and Validation process for modified IEEE 39-bus system.

The training and validation loss trends for the AE model are provided in Figs. 17, 18, 19, 20, 21 and 22. Figures 17 and 18 illustrates the RMSE progression during training and validation process for 9 bus and IEEE 39 bus system respectively, where initially, the RMSE is relatively high approx. 1.10, signifying notable discrepancies between the input and reconstructed data. However, as training progresses and iterations increase, the RMSE steadily declines and reaches to minimum value of approx. 0.4371, indicating the model’s ability to learn and adapt to normal operational conditions effectively. MAE during training and validation process has been presented in Figs. 19 and 20 for 9 bus and IEEE 39 bus system respectively. The starting value of MAE for 9-bus system is 0.7809 and after completion of training the value reduces to 0.2965, whereas for IEEE-39 bus system MAE started at 159.45, a very high value but gradually decreases to 10.06, which shows that the model can learn and adopt the system conditions. Figures 21 and 22 displays the loss during the training and validation phase for 9 bus and IEEE 39 bus system respectively, further supporting the model’s robustness. The value started from 1.2050 and rested at 0.2148 for 9-bus system and so for IEEE-39 bus the values are 1.2343 to 0.1853. The gradual reduction in validation loss confirms that the AE model consistently performs well, even under slightly abnormal conditions such as component outages. The loss function in this proposed model is Mean Square Error (MSE). Overall, the findings presented in this study highlight the AE model’s effectiveness in anomaly detection by analysing reconstruction error. The clear distinction between normal and anomalous data, as reflected in the MAE and RMSE metrics, validates the model’s precision. Moreover, the model’s capability to handle both small-scale and large-scale datasets underscores its potential for real-world applications within VPP systems. The AE model’s ability to accurately detect cyber-attacks on critical system parameters ensures the secure and reliable operation of VPPs. All these performance metric of this work during training and validation process of both proposed system is given in Table 3.

**Table 3:** Comparision of loss, Rmse and Mae (this work).

Iteration	Loss (9 Bus)	RMSE (9 Bus)	MAE (9 Bus)	Loss (IEEE-399 Bus)	RMSE (IEEE-39 Bus)	MAE (IEEE-39 Bus)
0	1.2050	1.1003	0.7809	1.2343	1.0992	159.4541
50	0.3547	0.5894	0.3956	0.3541	0.5801	132.9261
100	0.3075	0.5517	0.3713	0.3064	0.5518	100.7124
150	0.2890	0.5301	0.3620	0.2813	0.5352	95.5504
200	0.2795	0.5107	0.3549	0.2708	0.5225	91.9748
250	0.2683	0.5052	0.3494	0.2626	0.5116	87.5240
300	0.2617	0.4932	0.3461	0.2581	0.5098	79.6287
350	0.2563	0.4860	0.3400	0.2467	0.4974	26.0389
400	0.2544	0.4788	0.3380	0.2362	0.4976	16.0382
450	0.2416	0.4840	0.3340	0.2304	0.4883	13.4985
500	0.2357	0.4798	0.3311	0.2215	0.4836	12.6734
550	0.2361	0.4703	0.3261	0.2205	0.4780	11.9068
600	0.2301	0.4724	0.3182	0.2039	0.4697	12.3451
650	0.2326	0.4554	0.3134	0.1967	0.4713	12.0025
700	0.2232	0.4407	0.3077	0.1944	0.4650	11.8378
750	0.2167	0.4398	0.3068	0.1899	0.4643	10.6641
800	0.2176	0.4271	0.3030	0.1907	0.4616	9.7745
840	0.2148	0.4371	0.2965	0.1853	0.4593	10.0616

**Fig. 23:**
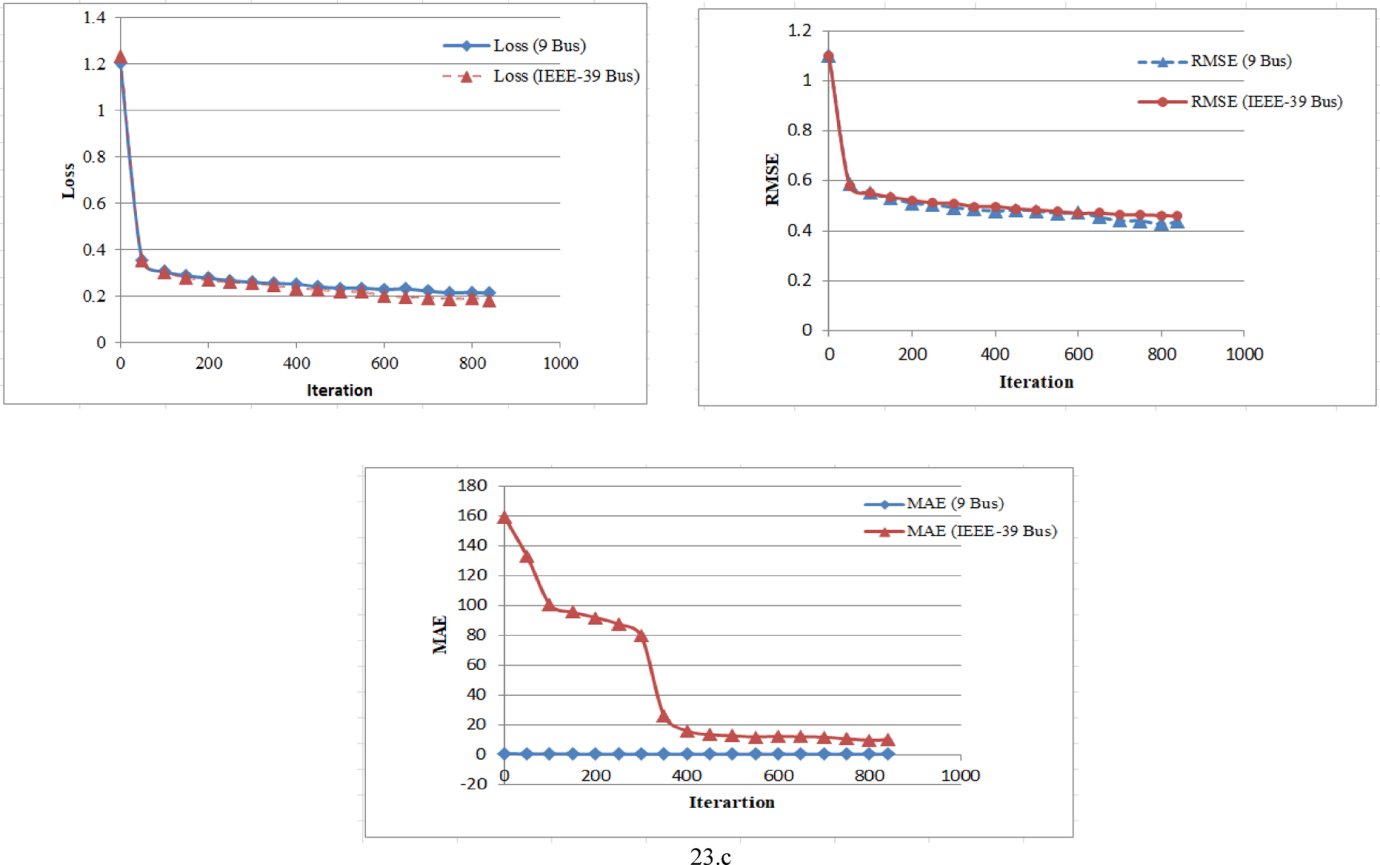
Performance comparison for 9-bus and IEEE-39 bus system (a, b, c)

The Table 3 shows the trends for loss, RMSE and MAE for proposed model during validation process. For both the 9-bus and IEEE 39-bus systems, the loss consistently decreases over iterations. Also RMSE and MAE show steady declines, indicating improved reconstruction accuracy. The most significant improvements occur in the first 300–400 iterations, after which the changes become more gradual. The IEEE 39-bus system shows a dramatic drop in MAE, suggesting that the model effectively learns to reconstruct signals despite the system’s complexity. The final values indicate the model has converged well, with only marginal improvements after 700 iterations. The graphical visualization for this comparison is given in Fig. 23.

The Table 4 presents a performance comparison of different machine learning models based on MAE and RMSE across various studies and datasets. From the table this can be concluded that traditional ML models such as Random Forest, MLP, and CNN from^52^ exhibit relatively low MAE and RMSE values, with the CNN-LSTM hybrid model achieving the best performance (MAE: 0.38, RMSE: 2.40). Study^53^ evaluates different regression models using the Subflow Bwd Byts feature. Linear Regression and SMOreg show competitive performance with low RMSE, whereas LSTM exhibits significantly higher errors (MAE: 31.99, RMSE: 35.04), indicating poor performance in this context. This work evaluates an unsupervised AE model on two different VPP system datasets (9-bus and IEEE 39-bus). The MAE values (13.65 and 13.10, respectively) are higher than other models, but the RMSE values (0.6041 and 0.6719) are among the lowest, suggesting that while absolute errors are higher, the overall model stability and consistency may be better. This work can be further extended for optimizing the value of MAE with some hybrid models.

**Table 4:** Performance comparison with diffeernet algorithm.

Reference	Algorithm / ML Model	MAE	RMSE
^52^	Random Forest	0.57	2.04
	MLP	0.69	2.58
	CNN	0.58	2.70
	LSTM	0.73	2.76
	CNN-LSTM	0.38	2.40
^53^ Subflow Bwd Byts	Linear Regression	0.718	0.8163
	SMOreg	0.7251	1.2969
	LSTM	31.9957	35.0395
[this work]	AE ( 9 bus)	13.65	0.6041
[this work]	AE (IEEE 39 bus)	13.10	0.6719

Autoencoders are neural networks trained to reconstruct input data. They learn a compressed representation of normal system behavior, making them well-suited for anomaly detection. The algorithm learns complex patterns in high-dimensional data, can generalize well to unseen attack patterns and effective for nonlinear relationships in power systems. On the other hand AE requires a large amount of normal training data, sensitive to hyperparameter tuning and computationally expensive compared to simpler methods. Some of the traditional methods like State Estimation-Based Residual Analysis, Principal component Analysis (PCA), clustering based methods are also being proposed for anomaly detection. The basic comparison is given in Table 5.

**Table 5:** Comparison with traditional/relevant methods.

Method	Strength	Weakness	MAE/RMSE Performance
Autoencoder ^47,48,54^, [this work]	Learns nonlinear patterns, good generalization	Requires large training data	Lower for normal data, higher for FDIA
State Estimation Residuals ^55^	Fast, interpretable	Bypassed by sophisticated attacks	Small errors for stealthy attacks
PCA ^56^	Simple, efficient	Limited for nonlinear systems	Higher for anomalies, but less robust
Clustering (K-Means, DBSCAN) ^32,57^	Works without labeled data	Parameter-sensitive	Varies with cluster separation
Isolation Forest / One-Class SVM ^58^	No need for normal data	Sensitive to hyperparameters	MAE/RMSE may be inconsistent

Therefore, Autoencoder-based methods show promise for FDIA detection, especially in nonlinear systems whereas Residual analysis remains effective but can be bypassed. PCA and clustering work well for structured data but struggle with stealthy attacks, and anomaly detection models (e.g., Isolation Forest, One-Class SVM) require careful tuning.

### Impacts of cyber attacks

Cyber-attacks on VPP can have far-reaching consequences, affecting the reliability, security, and efficiency of energy systems. The affects can be in the form of financial losses, system reliability and stability, energy market disruption, impacts on consumer and prosumers, operations disruptions, energy security risks and may have long term impacts such as reduced in system efficiency and blackout. FDIA can manipulate bid prices and energy quantities, leading to unfair advantages or losses in energy markets, which comes under the scope of this work. There will be revenue loss as consequences of alterations in energy pricing or generation data can cause losses for VPP operators, prosumers, and energy traders.

**Fig. 24:**
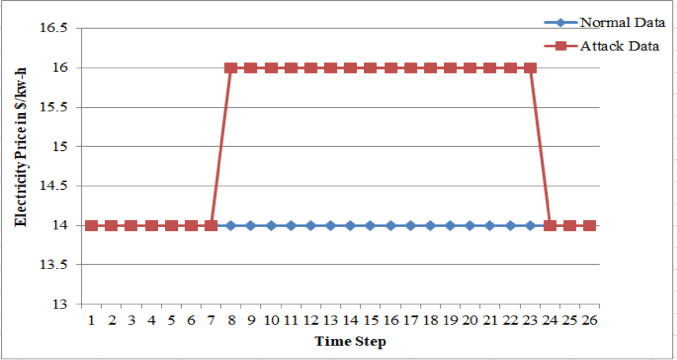
False data injection attack on Electricity price within the limits.

**Fig. 25:**
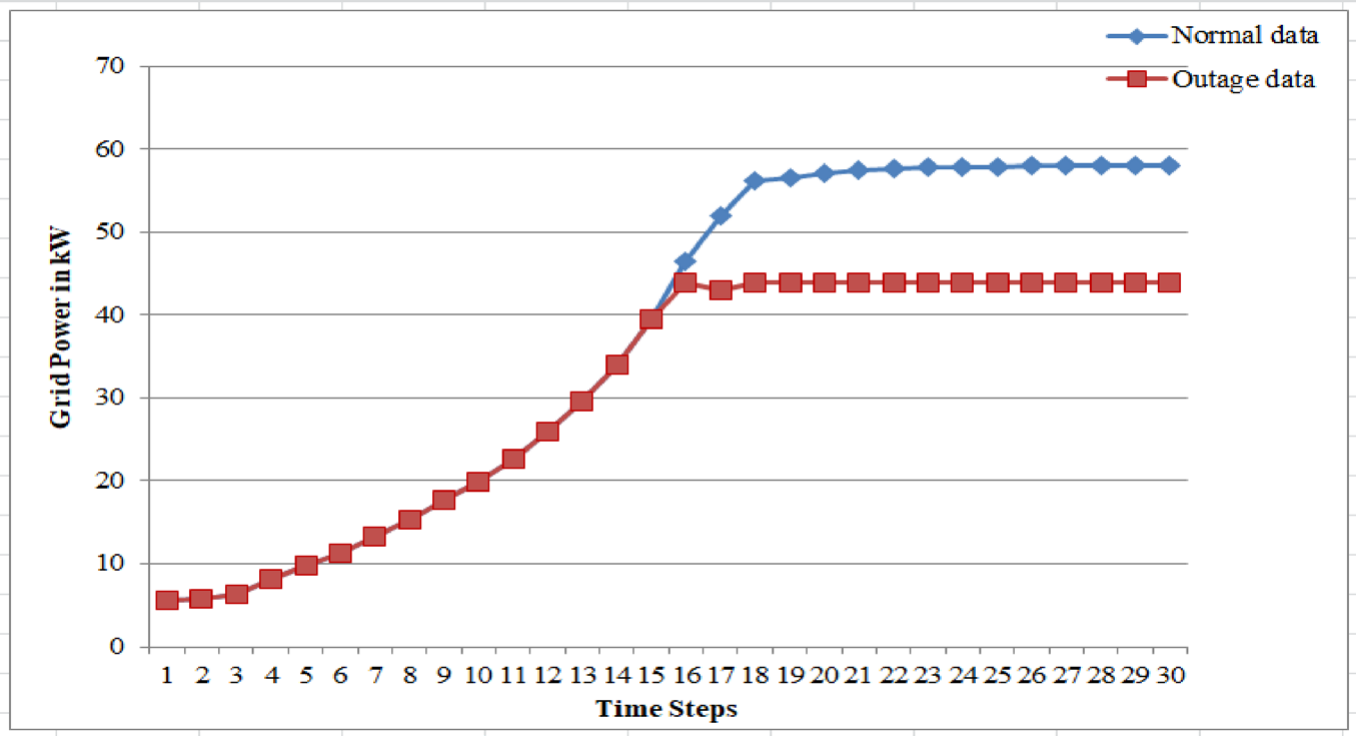
Sample for one generator outage for small range.

In this work, FDIA particularly in the form of anomalies in VPP system has been analyzed for two critical aspects: (1) PV power and grid power, which are the bid quantities submitted by the generating sources, and (2) electricity price fluctuations. Cyber attackers can target the system with the aim of disrupting its reliability by significantly altering bid values to a level far beyond the defined bidding range. Such manipulations can result in major deviations in the LMP of the energy market, leading to a cascade of effects on the overall market performance. Alternatively, if the attackers’ primary goal is financial gain within the energy market, the variation in bid values will likely remain within the allowable limits of the bidding range. By subtly manipulating electricity prices within a narrow range of $14/kWh to $16/kWh, as shown in Figs. 24 and Table 6 a, attackers can achieve financial gains while remaining undetected by traditional monitoring systems. Although the average deviation from the actual electricity price is just 0.48%, such minor fluctuations can cumulatively lead to significant financial impacts over time. Moreover, generator outage data for similarly small variations, illustrated in Fig. 25 and sample data is provided in Table 6b, and demonstrate how even minimal disruptions can have cascading effects on market operations and grid stability. These findings highlight the need for advanced anomaly detection systems and proactive measures to mitigate the risks associated with such stealthy attacks, which, if left unaddressed, can erode market integrity and consumer trust. In the presented scenario, one of the generator units is non-operational, and the power generation from the remaining three units is fixed at 44 kW for a very short time interval, which deviates from the system’s normal operating conditions. As illustrated in the figure, only a single sample data point is shown, with the deviation in power generation kept within 4% of the original output. Even small fluctuations in power output can create ripple effects across the system, influencing load demand patterns, electricity pricing, and overall power supply dynamics. These variations can introduce operational challenges, such as frequency and voltage instability, which may impose additional tariffs on consumers or lead to revenue losses for service providers. Strengthening cyber security frameworks can help safeguard critical energy infrastructure against sophisticated cyber threats and maintain consumer trust in VPP operations.

Root cause analysis of cyber-attacks is critical for the smooth and reliable operation of the energy market. Identifying the source of disruptions allows operators to implement the appropriate corrective measures. However, if the issue is identified as a cyber-attack, immediate action is required to prevent it from impacting the overall performance of the system. One effective measure to counter cyber intrusions is the implementation of packet filtering mechanisms. These can block unauthorized access by filtering network packets associated with specific IP addresses in the system, thereby mitigating potential cyber threats. Such solutions are crucial for maintaining system integrity. Any cyber-attack targeting prosumers demands special attention and more detailed analysis. Prosumers are essential to the bidirectional flow of energy and information, making them particularly vulnerable and critical in ensuring the stability of the VPP system.

While this work focuses on FDIA affecting the VPP system as a whole, it does not specifically analyses cyber intrusions targeting individual consumers or prosumers. This represents an important area for future research. Exploring the implications of cyber-attacks on prosumers and their role in VPP systems will enhance the understanding of vulnerabilities and inform the development of more comprehensive cyber security strategies. Detecting cyber intrusions in the VPP system remains a crucial challenge for ensuring safe, secure, and dependable operations. It is essential to maintain the CIA triad—Confidentiality, Integrity, and Availability—to secure the energy market against cyber threats and ensure the resilience of modern power systems. By successfully reconstructing normal data and identifying anomalies with minimal error, the proposed AE model proves to be a robust tool for detecting cyber intrusions in VPP systems. This approach highlights the importance of leveraging advanced ML techniques to enhance the security and resilience of modern power systems.

**Table 6:** Sample data for false data in a) electricity price and b) generator outage

a	
Normal Data	Attack Data
14	14
14	14
14	14
14	14
14	14
14	14
14	14
14	16
14	16
14	16
14	16
14	16
14	16
14	16
14	16
14	16
14	16
14	16
14	16
14	16
14	16
14	16
14	16
14	14
14	14

b	
Normal data	Outage data
9.738186	9.7381859
11.33506	11.335059
13.34243	13.342432
15.28133	15.281328
17.62124	17.62124
19.86142	19.861419
22.69559	22.695592
25.88921	25.889212
29.58706	29.587063
34.00579	34.00579
39.53797	39.537972
46.55902	44
51.97144	43
56.1438	44
56.66677	44
57.17026	44
57.45084	44
57.72538	44
57.88166	44
57.89718	44
57.91268	44
57.98988	44
57.99625	44
58.00261	44
58.00285	44

### Limitation and future work

The proposed model has certain shortcomings that point to potential directions for future research, notwithstanding the advancements and successes of this study. For instance, the model works well on some datasets but have trouble with time-series data that has a lot of noise. Furthermore, it lacks flexibility in how it handles time slice durations. Although the performance of the model is evaluated by MAE and RMSE, this can be further extended to a complete set of evaluation metrics with binary variable as: recall, precision, confusion matrix, F1-score and accuracy. The limitation and future work of this study can be highlighted as:

- Feature extraction: The feature from the attack scenario and component outage has been considered to attack condition. So the algorithm can be further exploited for extracting these system features.
- Evaluation metric: A logistic regression classification can be used for time series evaluation and can extend the performance metric with accuracy, precision and F1-score for helping to assess its predictive effectiveness.
- Hybrid ML models: This work can be further extended to increase the model effectiveness, accuracy and to reduce the errors by integrated with other ML model like CNN or LSTM. By adopting a hybrid approach, the strengths of traditional machine learning models and deep learning architectures can be leveraged to enhance predictive performance and minimize errors in time series classification tasks.

## Conclusions

In this work, an unsupervised Autoencoder machine learning approach has been presented to detect network-based cyber-attacks in the VPP system. The VPP system was developed and simulated in MATLAB, and the proposed detection model was implemented and investigated within the same environment. This approach focuses on detecting FDIA, particularly the alteration of bid quantities and electricity prices, which are critical to the operation and reliability of the VPP system. Two cases have been taken namely, 9 bus system and IEEE-39 bus system. The output power of the Solar Photovoltaic system and conventional grid power are taken as the bidding quantities of the VPP system. These quantities represent the energy supply offered to the market, making them key targets for potential cyber-attacks. The AE model successfully detected various attack scenarios, including: manipulation of bidding quantities grid power and PV power and false data injection in electricity prices in the energy market. The model employs a low-dimensional hidden layer in the AE architecture, where the decoder reconstructs the output layer from the encoded representations, preserving critical information while reducing complexity. The AE model demonstrates its capability to detect anomalies in time-series data, which is essential for identifying patterns and trends in dynamic systems like VPPs. The developed AE model is versatile and can be extended to handle various anomalies, ensuring scalability for more complex VPP systems. The detection model reliably identified cyber-attacks with high accuracy and low reconstruction error. The model’s performance metrics, Mean Absolute Error and Root Mean Square Error, confirmed its robustness in detecting anomalies while maintaining computational efficiency. There are opportunities for further development such as; the current methodology can be applied to larger, more complex VPP systems with additional components such as multiple energy storage units, wind turbines, and dynamic load conditions. In addition to anomaly detection, future research can focus on root-cause analysis to identify the source of cyber-attacks, enabling quicker mitigation and recovery. Future research and development in this area can significantly contribute to building more effective, reliable, and cyber-secure energy markets.

## Data Availability

The datasets used and/or analysed during the current study available from the corresponding author on reasonable request.

## References

[1] Abdolrasol, G. M., Hannan, M. & Hussain, M. A. Energy management scheduling for microgrids in the virtual power plant system using artificial neural networks. *Energies* **14**, 6507 (2021).

[2] Wang, X. et al. A review on virtual power plant concept, application and challenges 2019 IEEE PES innovative smart grid technologies Asia.

[3] Etherden, N., Vyatkin, V. & Bollen, M. H. J. Virtual power plant for grid services using IEC 61850. *IEEE Trans. Industr. Inf.* **12** (1), 437–447 (February 2016).

[4] Gunduz, M. Z. & Das, R. Cyber-security on smart grid: threats and potential solutions. *Comput. Netw.*10.1016/j.comnet.2019.107094 (2019).

[5] Ustun, T. S. & Hussain, S. M. S. A Review of Cybersecurity Issues in Smartgrid Communication Networks, 2019 International Conference on Power Electronics, Control and Automation (ICPECA), New Delhi, India, pp. 1–6. (2019).

[6] Zhang, H., Liu, B. & Wu, H. Smart Grid Cyber-Physical Attack and Defense: A Review, in IEEE Access, vol. 9, pp. 29641–29659, (2021). 10.1109/ACCESS.2021.3058628

[7] Tan, S., Song, W., Huang, D., Dong, Q. & Tong, L. Distributed software emulator for Cyber-Physical analysis in smart grid. *IEEE Trans. Emerg. Top. Comput.* **5** (4), 506–517. 10.1109/TETC.2014.2364928 (2017).

[8] Chawla, A. et al. Cyber–physical testbed for wide area measurement system employing IEC 61850 and IEEE C37. 118 based communication. *Energy Rep.* **8**, 570–578 (2022).

[9] Saxena, N., Xiong, L., Chukwuka, V. & Grijalva, S. Impact Evaluation of Malicious Control Commands in Cyber-Physical Smart Grids, in IEEE Transactions on Sustainable Computing, vol. 6, no. 2, pp. 208–220, 1 April-June (2021). 10.1109/TSUSC.2018.2879670

[10] Kaur, K., Kaddoum, G. & Zeadally, S. Blockchain-Based Cyber-Physical Security for Electrical Vehicle Aided Smart Grid Ecosystem, in IEEE Transactions on Intelligent Transportation Systems, vol. 22, no. 8, pp. 5178–5189, Aug. (2021). 10.1109/TITS.2021.3068092

[11] Venkatachary, S. K., Prasad, J., Samikannu, R., Alagappan, A. & Andrews, L. J. B. Cybersecurity infrastructure challenges in IoT based virtual power plants. *J. Stat. Manage. Syst.* **23** (2), 263–276. 10.1080/09720510.2020.1724625 (2020).

[12] Buchta, R., Heine, F. & Kleiner, C. Challenges and Peculiarities of Attack Detection in Virtual Power Plants : Towards an Advanced Persistent Threat Detection System, 2022 IEEE 29th Annual Software Technology Conference (STC), Gaithersburg, MD, USA, pp. 69–81, (2022). 10.1109/STC55697.2022.00019

[13] Venkatachary, S. K., Alagappan, A. & Andrews, L. J. B. Cybersecurity challenges in energy sector (virtual power plants) - can edge computing principles be applied to enhance security? *Energy Inf.* **4**, 5. 10.1186/s42162-021-00139-7 (2021). 10.1186/s42162-021-00139-7 PMC8010494 35224445

[14] Gkoktsis, G., Lauer, H. & Jaeger, L. Risk Assessments in Virtual Power Plants with NESCOR Criteria, Practical Application, Advantages and Disadvantages, 18th International Conference on Availability, Reliability and Security (ARES 2023), August 29–September 01, 2023, Benevento, Italy. ACM, New York, NY, USA 11 Pages. 10.1145/3600160.3605179

[15] Khan, M., Hosseinzadehtaher, M. B., Shadmand, S. K. & Mazumder Cybersecurity Analytics for Virtual Power Plants, 2021 IEEE 12th International Symposium on Power Electronics for Distributed Generation Systems (PEDG), pp. 1–5, (2021). 10.1109/PEDG51384.2021.9494255

[16] Zhong, X., Jayawardene, I., Venayagamoorthy, G. K. & Brooks, R. Denial of Service Attack on Tie-Line Bias Control in a Power System With PV Plant, in IEEE Transactions on Emerging Topics in Computational Intelligence, vol. 1, no. 5, pp. 375–390, Oct. (2017). 10.1109/TETCI.2017.2739838

[17] Xu, Y. Risk of Jamming Attacks on a Virtual Power Plant with Multiple Distributed Generators, 2019 29th Australasian Universities Power Engineering Conference (AUPEC), Nadi, Fiji, 2019, pp. 1–6. 10.1109/AUPEC48547.2019.211918

[18] Pan, S., Morris, T. H. & Adhikari, U. A specification-based intrusion detection framework for cyber-physical environment in electric power system. *IJ Netw. Secur.* **17** (2), 174–188 (2015).

[19] Chen, J., Yan, J., Du, H., Debbabi, M. & Kassouf, M. Vulnerability Analysis of Virtual Power Plant Voltage Support under Denial-of-Service Attacks, 2023 IEEE Power & Energy Society Innovative Smart Grid Technologies Conference (ISGT), Washington, DC, USA, pp. 1–5, (2023). 10.1109/ISGT51731.2023.10066361

[20] Ozay, M., Esnaola, I., Vural, F. T. Y., Kulkarni, S. R. & Poor, H. V. Machine learning methods for attack detection in the smart grid. *IEEE Trans. Neural Networks Learn. Syst.* **27** (8), 1773–1786 (2016). 10.1109/TNNLS.2015.2404803 25807571

[21] Xiang, Z., Guangyu, H. & Zhigong, W. Masquerade detection using support vector machines in the smart grid, in Computational Sciences and Optimization (CSO), 2014 Seventh International Joint Conference on. IEEE, pp. 30–34. (2014).

[22] Taheri, S. I., Davoodi, M. & Ali, M. H. Mitigating cyber anomalies in virtual power plants using Artificial-Neural-Network-Based secondary control with a federated Learning-Trust adaptation *Energies* 17 2024, no. **3**: 619. 10.3390/en17030619

[23] Chu, T., Yan, Z., Gong, X. & Dong, F. Network Attack Detection Method for Distributed Economic Dispatch of Virtual Power Plants, 2022 First International Conference on Cyber-Energy Systems and Intelligent Energy (ICCSIE), Shenyang, China, 2023, pp. 1–6. 10.1109/ICCSIE55183.2023.10175256

[24] Xie, L., Mo, Y. & Sinopoli, B. False Data Injection Attacks in Electricity Markets, 2010 First IEEE International Conference on Smart Grid Communications, Gaithersburg, MD, USA, pp. 226–231, (2010). 10.1109/SMARTGRID.2010.5622048

[25] Ahmed, M. & Pathan, A. S. K. False data injection attack (FDIA): an overview and new metrics for fair evaluation of its countermeasure. *Complex. Adapt. Syst. Model.* **8**, 4. 10.1186/s40294-020-00070-w (2020).

[26] Habib, A. et al. False data injection attack in smart grid cyber physical system: issues, challenges, and future direction. *Comput. Electr. Eng.*, 107, 2023, 108638, ISSN 0045-7906, 10.1016/j.compeleceng.2023.108638

[27] Roomi, M. M., Hussain, S. M. S., Mashima, D. & Chang, E. C. Analysis of False Data Injection Attacks Against Automated Control for Parallel Generators in IEC 61850-Based Smart Grid Systems, in IEEE Systems Journal, vol. 17, no. 3, pp. 4603–4614, Sept. (2023).

[28] Xu, R. et al. Achieving Efficient Detection Against False Data Injection Attacks in Smart Grid, in IEEE Access, vol. 5, pp. 13787–13798, (2017). 10.1109/ACCESS.2017.2728681

[29] Hu, P. et al. Detection of false data injection attacks in smart grids based on expectation maximization. *Sensors* **23**, 1683. 10.3390/s23031683 (2023). 36772723 10.3390/s23031683 PMC9919858

[30] Unsal, D. B. Enhancing cybersecurity in smart grids: false data injection and its mitigation. *Energies* **14**, 2657 (2021).

[31] Shen, K., Yan, W., Ni, H. & Chu, J. Localization of false data injection attack in smart grids based on SSA-CNN. *Information* **14**, 180. 10.3390/info14030180 (2023).

[32] Tightiz, L., Nasimov, R. & Nasab, M. A. Implementing AI Solutions for Advanced Cyber-Attack Detection in Smart Grid, *International Journal of Energy Research*, 6969383, 21 pages, 2024. (2024). 10.1155/2024/6969383

[33] Aoufi, S., Derhab, A. & Guerroumi, M. Survey of false data injection in smart power grid: attacks, countermeasures and challenges. *J. Inform. Secur. Appl.*, 54, 2020, 102518, ISSN 2214 – 2126, 10.1016/j.jisa.2020.102518

[34] Zhang, Y. Cyber Physical Security Analytics for Transactive Energy Systems, in IEEE Transactions on Smart Grid, vol. 11, no. 2, pp. 931–941, March (2020). 10.1109/TSG.2019.2928168

[35] Ustun, T. S., Hussain, S. M. S., Yavuz, L. & Onen, A. Artificial Intelligence Based Intrusion Detection System for IEC 61850 Sampled Values Under Symmetric and Asymmetric Faults, in IEEE Access, vol. 9, pp. 56486–56495, (2021). 10.1109/ACCESS.2021.3071141

[36] Ustun, T. S. et al. Machine Learning-Based intrusion detection for achieving cybersecurity in smart grids using IEC 61850 GOOSE messages. *Symmetry* **13** (5), 826. 10.3390/sym13050826 (2021).

[37] Mavikumbure, H. S., Wickramasinghe, C. S., Marino, D. L., Cobilean, V. & Manic, M. Anomaly Detection in Critical-Infrastructures using Autoencoders: A Survey, IECON 2022–48th Annual Conference of the IEEE Industrial Electronics Society, Brussels, Belgium, pp. 1–7, (2022). 10.1109/IECON49645.2022.9968505

[38] Li, P., Liu, Y., Xin, H. & Jiang, X. A Robust Distributed Economic Dispatch Strategy of Virtual Power Plant Under Cyber-Attacks, in IEEE Transactions on Industrial Informatics, vol. 14, no. 10, pp. 4343–4352, Oct. (2018). 10.1109/TII.2017.2788868

[39] Venkatachary, S. K. et al. Cybersecurity and cyber-terrorism challenges to energy-related infrastructures – Cybersecurity frameworks and economics – Comprehensive review. *Int. J. Crit. Infrastruct. Prot.* **45**, 1874–5482. 10.1016/j.ijcip.2024.100677 (2024).

[40] Lin, J., Yu, W., Yang, X., Xu, G. & Zhao, W. On false data injection attacks against distributed energy routing in smart grid, in Proceedings of the IEEE/ACM Third International Conference on Cyber Physical Systems. IEEE, 2012. (2012).

[41] Gkoktsis, H. Assessing the cyber threat landscape for virtual power plants. *Latin-American J. Comput.* **9** (2), 1390–9266 (2022).

[42] https://www.cfr.org/cyber-operations/compromise-power-grid-eastern-ukraine

[43] kumar, V. S. & Narasimhan, V. L. Using Deep Learning For Assessing Cybersecurity Economic Risks In Virtual Power Plants, 2021 7th International Conference on Electrical Energy Systems (ICEES), Chennai, India, pp. 530–537, (2021). 10.1109/ICEES51510.2021.9383723

[44] Hou, B. Performance of Neighborhood-Watch-Based Resilient Distributed Energy Management Algorithm Under Different Types of Cyberattacks, 2021 IEEE 4th International Electrical and Energy Conference (CIEEC), Wuhan, China, 2021, pp. 1–5. 10.1109/CIEEC50170.2021.9510236

[45] Chen, Z., Yeo, C. K., Lee, B. S. & Lau, C. T. Autoencoder-based network anomaly detection, 2018 Wireless Telecommunications Symposium (WTS), Phoenix, AZ, USA, pp. 1–5, (2018). 10.1109/WTS.2018.8363930

[46] Torabi, H., Mirtaheri, S. L. & Greco, S. Practical autoencoder based anomaly detection by using vector reconstruction error. *Cybersecurity* **6**, 1 ,2023. 10.1186/s42400-022-00134-9

[47] Gensler, J., Henze, B., Sick, N., Raabe & LSTM Neural Networks,. Deep Learning for solar power forecasting — An approach using AutoEncoder and IEEE International Conference on Systems, Man, and Cybernetics (SMC), 2016, pp. 002858–002865, (2016). 10.1109/SMC.2016.7844673

[48] Sagheer, M. & Kotb Unsupervised Pre-training of a deep LSTM-based stacked autoencoder for multivariate time series forecasting problems. *Sci. Rep.* **9** (19038). 10.1038/s41598-019-55320-6 (2019). 10.1038/s41598-019-55320-6 PMC6911101 31836728

[49] Kopčan, J., Škvarek, O. & Klimo, M. Anomaly detection using autoencoders and deep Convolution generative adversarial networks. *Transp. Res. Procedia*. **55**10.1016/j.trpro.2021.07.113 (2021). Pages 1296–1303, ISSN 2352 – 1465.

[50] Tsai, D. M. & Jen, P. H. Autoencoder-based anomaly detection for surface defect inspection. *Adv. Eng. Inform.* **48**10.1016/j.aei.2021.101272 (2021). 101272, ISSN 1474 – 0346.

[51] Zhang, L. et al. Time-Series neural network: A High-Accuracy time-Series forecasting method based on kernel filter and time attention. *Information* **14**, 500. 10.3390/info14090500 (2023).

[52] Bhusal, N., Gautam, M. & Benidris, M. Cyber-attack Detection on Distributed Frequency Control of Islanded MGs Using Machine Learning, 2021 IEEE Industry Applications Society Annual Meeting (IAS), Vancouver, BC, Canada, 2021, pp. 1–6. 10.1109/IAS48185.2021.9677432

[53] Ahmed, Y., Azad, M. A. & Asyhari, T. Rapid forecasting of cyber events using machine Learning-Enabled features. *Information* **15**, 36. 10.3390/info15010036 (2024).

[54] Zideh, M. J., Khalghani, M. R. & Solanki, S. K. An unsupervised adversarial autoencoder for cyber attack detection in power distribution grids. *Electr. Power Syst. Res.* **232**, 0378–7796. 10.1016/j.epsr.2024.110407 (2024).

[55] Zhu, Y. & Liu, R. Detection of false data injection attacks on power systems based on measurement-eigenvalue residual similarity test. *Front. Energy Res.* **11**, 1285317. 10.3389/fenrg.2023.1285317 (2023).

[56] Cui, J., Gao, B. & Guo, B. A novel detection and defense mechanism against false data injection attack in smart grids. *IET Gener Transm Distrib.* **17**, 4514–4524. 10.1049/gtd2.12848 (2023).

[57] An, H. et al. Cluster partition-fuzzy broad learning-based fast detection and localization framework for false data injection attack in smart distribution networks. *Sustainable Energy Grids Networks*. **40**, 2352–4677. 10.1016/j.segan.2024.101534 (2024).

[58] Song, Y., Yu, Z., Liu, X., Tian, J. & Chen, M. Isolation Forest based Detection for False Data Attacks in Power Systems, 2019 IEEE Innovative Smart Grid Technologies - Asia (ISGT Asia), Chengdu, China, 2019, pp. 4170–4174. 10.1109/ISGT-Asia.2019.8881319

